# Examination of Apoptosis Signaling in Pancreatic Cancer by Computational Signal Transduction Analysis

**DOI:** 10.1371/journal.pone.0012243

**Published:** 2010-08-19

**Authors:** Felix Rückert, Gihan Dawelbait, Christof Winter, Arndt Hartmann, Axel Denz, Ole Ammerpohl, Michael Schroeder, Hans Konrad Schackert, Bence Sipos, Günter Klöppel, Holger Kalthoff, Hans-Detlev Saeger, Christian Pilarsky, Robert Grützmann

**Affiliations:** 1 Department of Visceral, Thoracic and Vascular Surgery, University Hospital Carl Gustav Carus, Technical University Dresden, Dresden, Germany; 2 Bioinformatics Group, Biotechnological Centre, Technical University Dresden, Dresden, Germany; 3 Department of Pathology, University of Erlangen, Erlangen, Germany; 4 Division of Molecular Oncology, Clinic for General Surgery and Thoracic Surgery, Schleswig-Holstein University Hospitals, Kiel, Germany; 5 Department of Surgical Research, University Hospital Carl Gustav Carus, Technical University Dresden, Dresden, Germany; 6 Division of Molecular Oncology, Institute for Experimental Cancer Research, Schleswig-Holstein University Hospitals, Kiel, Germany; Health Canada, Canada

## Abstract

**Background:**

Pancreatic ductal adenocarcinoma (PDAC) remains an important cause of cancer death. Changes in apoptosis signaling in pancreatic cancer result in chemotherapy resistance and aggressive growth and metastasizing. The aim of this study was to characterize the apoptosis pathway in pancreatic cancer computationally by evaluation of experimental data from high-throughput technologies and public data bases. Therefore, gene expression analysis of microdissected pancreatic tumor tissue was implemented in a model of the apoptosis pathway obtained by computational protein interaction prediction.

**Methodology/Principal Findings:**

Apoptosis pathway related genes were assembled from electronic databases. To assess expression of these genes we constructed a virtual subarray from a whole genome analysis from microdissected native tumor tissue. To obtain a model of the apoptosis pathway, interactions of members of the apoptosis pathway were analysed using public databases and computational prediction of protein interactions. Gene expression data were implemented in the apoptosis pathway model. 19 genes were found differentially expressed and 12 genes had an already known pathophysiological role in PDAC, such as Survivin/BIRC5, BNIP3 and TNF-R1. Furthermore we validated differential expression of IL1R2 and Livin/BIRC7 by RT-PCR and immunohistochemistry. Implementation of the gene expression data in the apoptosis pathway map suggested two higher level defects of the pathway at the level of cell death receptors and within the intrinsic signaling cascade consistent with references on apoptosis in PDAC. Protein interaction prediction further showed possible new interactions between the single pathway members, which demonstrate the complexity of the apoptosis pathway.

**Conclusions/Significance:**

Our data shows that by computational evaluation of public accessible data an acceptable virtual image of the apoptosis pathway might be given. By this approach we could identify two higher level defects of the apoptosis pathway in PDAC. We could further for the first time identify IL1R2 as possible candidate gene in PDAC.

## Introduction

Pancreatic ductal adenocarcinoma (PDAC) is the 8th most common cancer in the western world [1]. Its mortality almost equals its incidence rate of 6.3/100,000 [2]. Despite combined modality therapy pancreatic carcinoma shows a unsatisfactory response to treatment [3]. Recently, a comprehensive genomic analysis of *Jones* et al. could identify apoptosis as a core signaling pathway in pancreatic cancer. The pathway was genetically altered in most of 24 primary pancreatic cancer cell lines [4]. Clinicopathologically, this defective apoptosis signaling contributes to the tumor's poor response to chemotherapy, ionizing radiation and immunotherapy [5] and affects the metastasizing capacity and growth rate of the tumor [6], [7]. Therefore, understanding of apoptosis resistance is a prerequisite for improving cancer therapy.

Apoptosis, or cell death program, can be activated by various mechanisms within the extrinsic and the intrinsic pathway. While activation of cell death receptors leads to the engagement of the extrinsic pathway, the intrinsic pathway is activated by mitochondria during cellular stress, both resulting in an activation of caspases [8].

**Figure 1:**
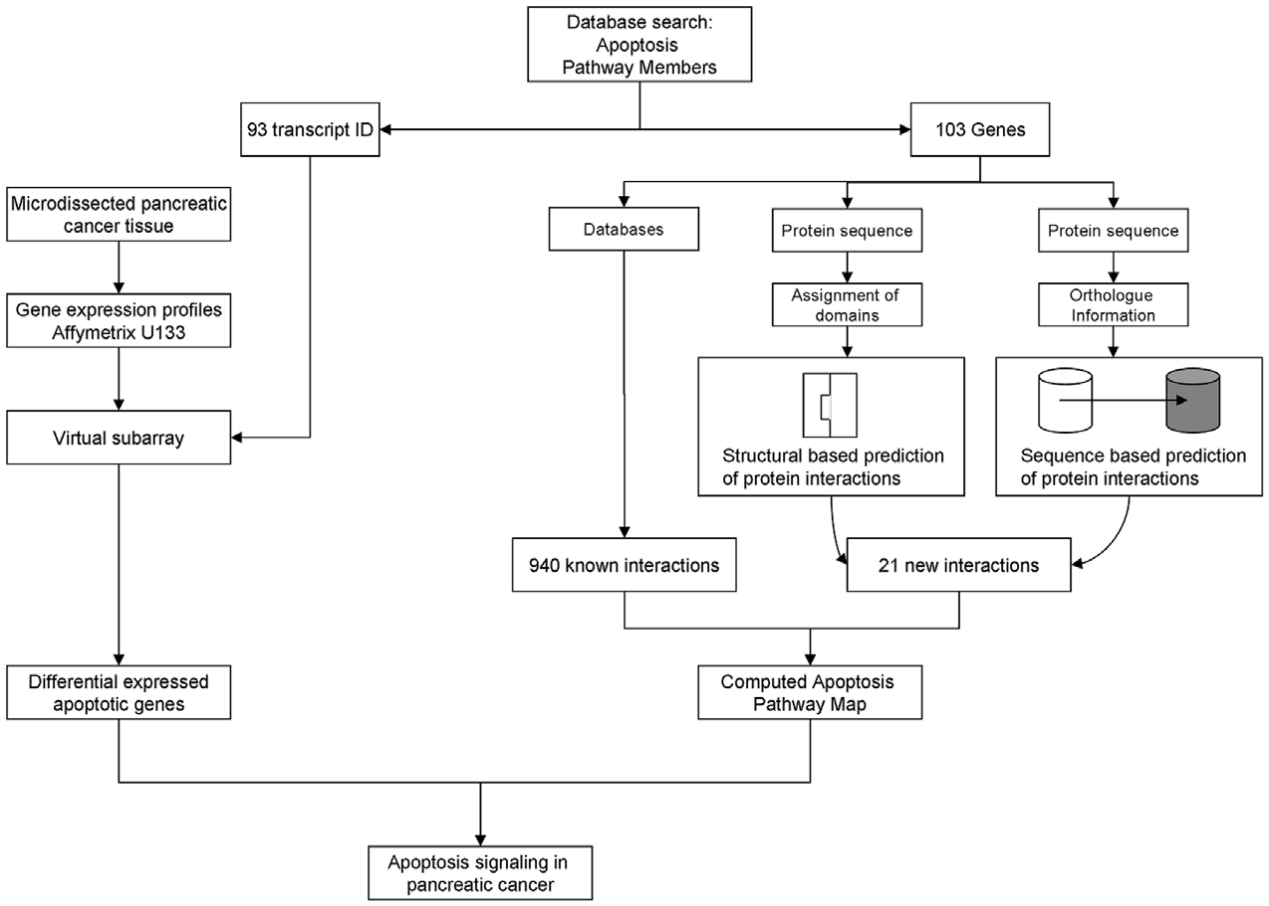
Graphic display of the study design.

Today, the apoptosis pathway is one of the best investigated intracellular pathways. However, interpretation of experimental data is hindered by the multitude of signaling molecules and complex interactions of the pathway. In this study we tried to approach the cell death pathway in pancreatic cancer by a computational analysis of experimental data from highthroughput technologies and public databases. We tried to use the great amount of information to model the intracellular information flow of the apoptosis pathway in pancreatic cancer. For a graphic display of the study design see **Figure 1**.

The implementation of gene expression data into a model of the apoptosis pathway obtained by protein interaction databases and protein interaction prediction showed a consistent pattern of higher-level defects in the intrinsic pathway and on the level of cell death receptors that can potentially result in the phenotype of apoptosis resistance in pancreatic cancer.

## Results

### Computational construction of the apoptosis pathway map

**Figure 2:**
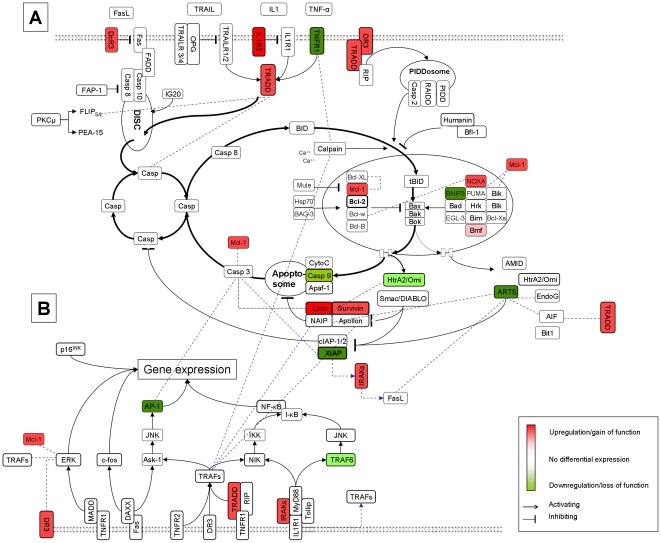
Pathway map of the apoptosis pathway. The nodes in these graphs represent receptors, ligands, effectors, kinases and transcription factors, while each edge describes a relation between these species. In the upper part of the figure the direct apoptosis induction is shown (**A**), whereas in the lower part the modulation through gene expression is depicted (**B**). Black interactions signify known protein interactions from databases. For better view we did not display all of the 940 known interactions, please see **File S3** for a list of all interactions. Blue edges signify computationally predicted interactions for all 103 apoptosis-associated genes with a high evidence level.

Interactions of the 103 apoptosis associated genes from our database search were initially evaluated by screening of protein-protein interaction databases. The search resulted in 940 known interactions. Those interactions represented experimentally proven interactions between defined proteins. This data was used to construct a pathway map, as mentioned above (**Figure 2**).

In a second step we tried to find previously unknown interactions between the 103 apoptosis-associated genes. We therefore had to assign structural families to the gene products because most of the structures were previously unknown. The structural assignment and family classification for the apoptotic associated genes resulted in the assignment of 53 genes. Applying the interface conservation evaluation to possible interactions between the products of those 53 genes resulted in 21 novel interactions (for examples see **File S2**, for whole data see **File S3**).

Those novel interactions represent putative interactions which are not yet experimentally proven. All new interactions were implemented in the first map of the cell death pathway (**Figure 2**).

### GeneChip results

**Figure 3:**
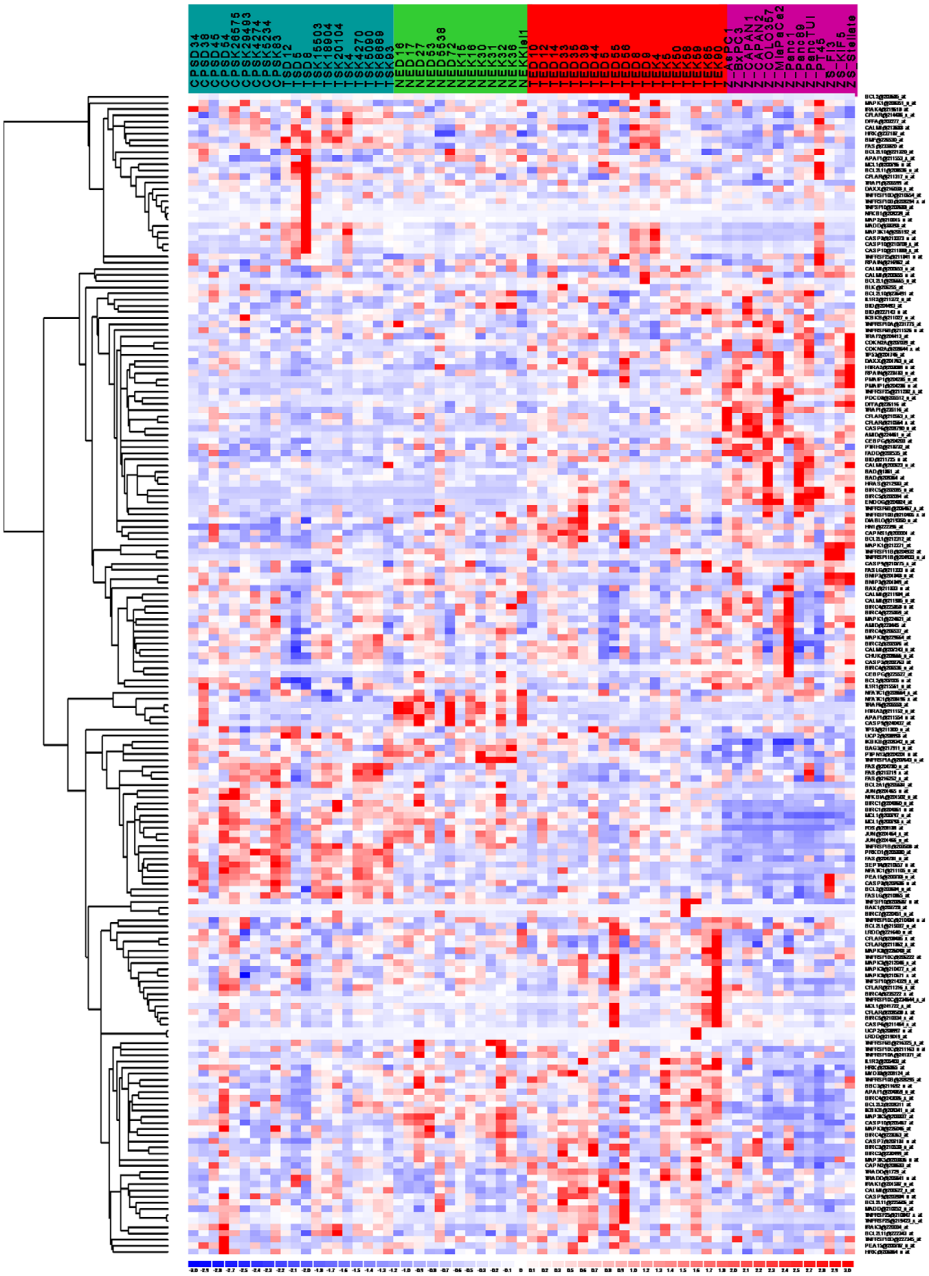
Analysis of apoptosis-associated gene expression in PDAC. Heat map of 19 microdissected PDACs (marked red), 13 samples of microdissected normal ductal cells (marked green), and 13 established pancreatic tumor cell lines (marked magenta) using the 93 differential gene set and a Euclidian distance matrix. Normal stromal cells served as internal quality control (marked blue).

We constructed a virtual subarray to identify gene expression changes of the 93 apoptotic genes for which an identifier could be obtained. To evaluate the performance of this approach we compared the results for the apoptotic gene set with whole genome and virtual subarray analysis. Of 23 probe sets identified with the virtual subarray analysis only 18 were detected in the whole genome analysis. The mean expression intensities of the probesets detected only by subarray analysis was 125 compared to 346 for probe sets detected with both methods, indicating that the virtual subarray analysis was more sensitive. The 23 probesets represented 19 differentially expressed genes (**Figure 3**). Of these, 11 genes were overexpressed and 8 were underexpressed in PDAC compared to microdissected normal ductal cells. Among the nineteen genes, 12 were already reported by other groups in PDAC (**Table 1**).

**Table 1:** Results of the GeneChip analysis.

No.	Affy ID	Name	Gene Symbol	Function	FC	Ref. in PDAC
	**Upregulated genes**					
**13**	205403_at	IL1-R2	IL1R2	Decoy-receptor	7.1	
**36**	202094_at 202095_s_at	Survivin/BIRC5	BIRC5	Apoptosis inhibitor	5.0	[19], [32]
**14**	219423_x_at 210847_x_at	DR3	TNFRSF25	Cell death receptor	4.1	[10]
**65**	204285_s_at 204286_s_at	NOXA	PMAIP1	MP	3.1	
**38**	220451_s_at	Livin/BIRC7	BIRC7	Apoptosis inhibitor	2.9	
**56**	241722_x_at	Mcl-1	MCL1	MP	2.8	[33], [34]
**18**	1729_at	TRADD	TRADD	Signal molecule	2.4	[35]
**21**	201587_s_at	Irak, pelle	IRAK1	Signal molecule	2.2	[35]
**10**	206467_x_at	DcR3	TNFRSF6B	Decoy receptor	2.1	[36], [37]
**72**	226530_at	BMF	BMF	MP	2.1	
**29**	220034_at	Irak, pelle	IRAK3	Signal molecule	2.0	[35]
	**Downregulated genes**					
**81**	210657_s_at	ARTS	SEPT4	MP	0.49	
**2**	201466_s_at	AP-1	JUN	Signal molecule	0.48	[38]
**35**	225858_s_at	XIAP	BIRC4	Apoptosis inhibitor	0.48	[19], [32]
**4**	207643_s_at	TNF-R1	TNFRSF1A	Cell death receptor	0.46	[37]
**68**	201848_s_at 201849_at	BNIP3	BNIP3	MP	0.37	[39], [40]
**45**	240437_at	Caspase 9	CASP9	Protease	0.27	[41]
**23**	205558_at	TRAF 6	TRAF6	Signal molecule	0.19	
**76**	211152_s_at	HtrA2/Omi	HTRA2	MP	0.13	

Upregulated genes (fold-change >2, q<5%) are listed in the upper part, the downregulated genes in the lower part of the table. The numbers represent the order of the genes in our supplementary data 2 (*MP = mitochondrial protein*; *FC = Fold change*).

### Verification of differential expression

**Figure 4:**
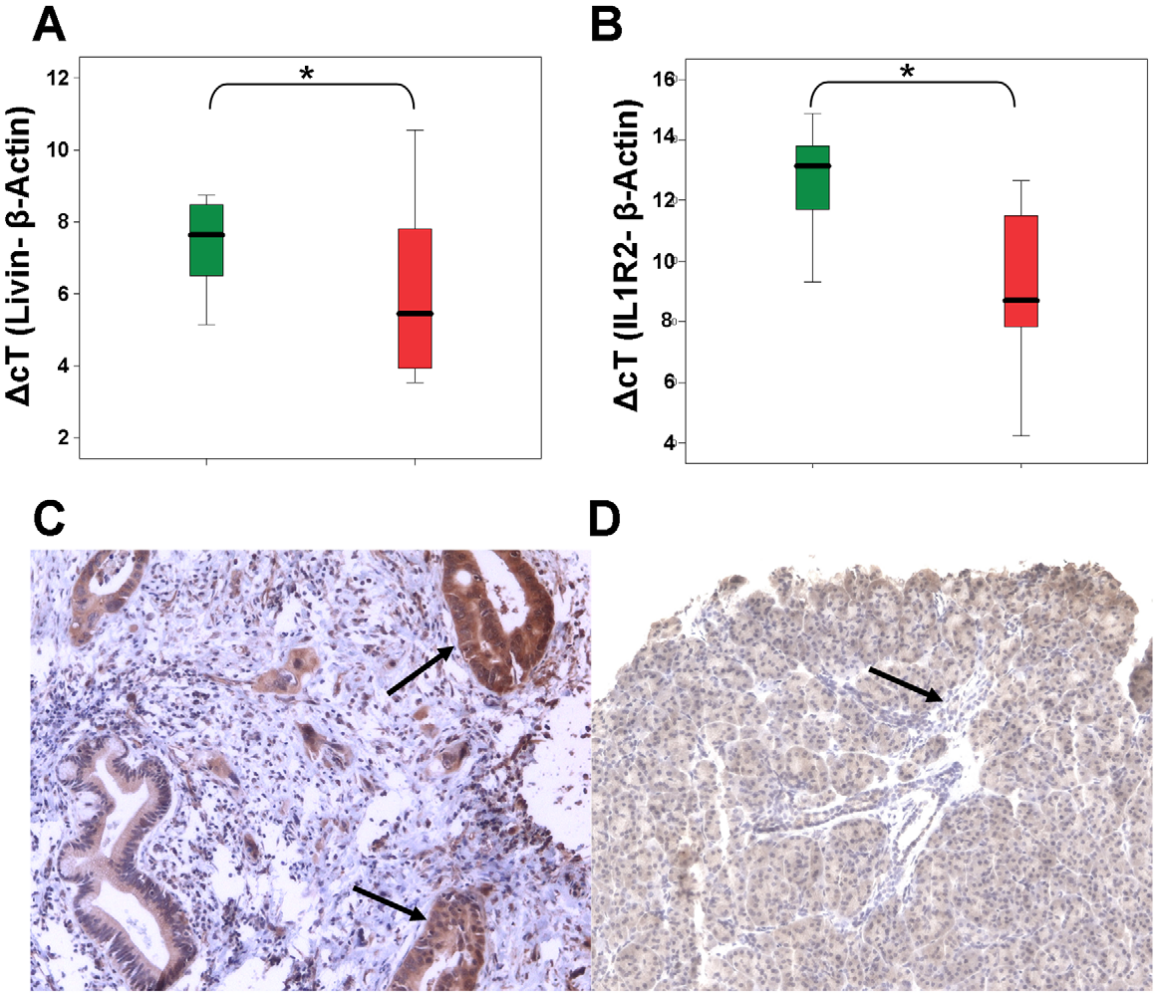
Validation of differential expressed genes by quantitative RT-PCR. The graphs display the results of the quantitative RT-PCR in normal tissue of the pancreas and pancreatic adenocarcinoma of Livin/BIRC7 (t-test with p = 0.01) (**A**) and IL1R2 (t-test with p = 0.035) (**B**). Immunohistochemical staining for Livin/BIRC7 in benign pancreas and invasive adenocarcinoma. Pancreatic carcinoma (arrow) showing intensive cytoplasmic staining (original magnification x100)(**C**). Benign ductal epithelium shows a noticeable fainter staining (arrow) (original magnification ×40)(**D**). * indicates p-value <0.05.

We selected two genes, Livin/BIRC7 and IL1R2, for validation by quantitative RT-PCR and/or immunohistochemistry. We confirmed an significant upregulation in PDAC cells or IL1R2 (*p* = 0.035) and LIVIN/BIRC7 (*p* = 0.01) (**Figure 4** **A,B**). Parallel to RT-PCR 16 samples from patients with PDAC and 16 normal pancreatic tissues were stained for Livin/BIRC7. 89% of the PDAC cells were tested positive for Livin/BIRC7, in contrast to only 62% of the of the normal ductal cells (*p* = 0.001). PDAC tissue showed also more intensive staining than normal tissue, and the results were statistically significant (*p*<0.001) (**Figure 4 C,D**).

## Discussion

Pancreatic cancer is a malignancy with very poor prognosis and no significant improvement in therapy over the last 30 years [1]. Recently, a comprehensive genomic analysis identified apoptosis as a core signaling pathway in pancreatic cancer [4].

The apoptosis pathway is one of the best investigated intracellular pathways. However, the pathway comprises a multitude of signaling molecules and displays complex interactions. This leaves results of experimental studies hard to interpret. In this study we tried to approach the cell death pathway in pancreatic cancer by a computational analysis of experimental data from high-throughput technologies and by evaluation of public databases. With the help of these technologies we tried to better understand the great amount of information and to make statements about disturbances in the information flow in the apoptosis pathway in pancreatic cancer. Our results were compared to previous publications on apoptosis in pancreatic cancer.

103 apoptosis associated genes were identified by database search. To assess interactions between the identified apoptosis associated genes we evaluated databases and computationally predicted protein interactions. The fact that apoptosis pathway is one of the best known pathways was mirrored by the great amount of experimental proven protein interactions in public databases. Our approach yielded 940 interactions and we could further identify 21 previously unknown interactions computationally by the sequence-based and structure-based prediction of protein interactions. Especially MCL-1, CASP 3, TRADD, SEPT 4, AIF, CALM and TRAF 6 showed branchings in the signaling cascade previously unknown. Although we could not experimentally proof those interactions, this is an evidence for the complexity of the information flow within this pathway. By setting cell death receptors as starting point for the signaling pathway we constructed a model of the pathway to visualize the interactions.

Gene expression analysis of the apoptosis-associated genes was performed using a virtual subarray in a set of microdissected tissue from normal pancreatic ducts and PDAC. Comparing the data from the subarray with whole genome analysis revealed considerably more probe sets to be differentially expressed using the virtual subarray approach. Interestingly the probe sets not identified by whole genome analysis displayed lower expression values, demonstrating that the construction of a virtual subarray might result in an enhanced sensitivity for detection at the lower end of gene expression intensities. This is mainly due to the smaller number of probe sets tested, reducing the possible noise of fluctuation during the analysis.

Gene expression analysis showed 19 differentially expressed genes. Of these, 11 genes were overexpressed and 8 were underexpressed in PDAC compared to microdissected normal ductal cells. Among the nineteen genes, 12 were already reported by other groups in PDAC.

Survivin, Livin, MCL-1, and DcR3 were upregulated and showed very good accordance to previous reports (see **File S1**). TNF-R1, BNIP3, and Caspase 9 were downregulated, those genes also showed good accordance to previous studies (see **File S1**).

However, XIAP, a member of the IAP family of proteins was downregulated in our analysis contrary to previous studies. This discrepancy might be due to tumor heterogeneity, a fundamental facet of all solid tumors [9]. It might also be due to the differences in study designs, because most of the previous studies on XIAP used pancreatic carcinoma cell lines, while we used microdissected native tumor tissue.

However, our data yielded interesting result, as we found two major foci of dysregulations within the cell death pathway.

One of the major foci was at the level of cell receptors. Generally, there seems to be a downregulation of cell death receptors, and an upregulation of decoy-receptors. The downregulation of the cell death receptor TNRF-1 and the upregulation of the Fas-decoy receptor DcR3 in our data was already reported earlier [10]. This dysregulation is meant to help the tumor evade the immune system, because of the diminished sensibility towards apoptotic ligands [11], [12].

Another decoy receptor, IL1R2, was chosen for further validation, because upregulation of this receptor was not reported previously. By means of quantitative RT-PCR we could for the first time validate an upregulation of IL1R2 in pancreatic cancer. IL1, the ligand of IL1R2 is known to be secreted by pancreatic cancer cells [13]. It has important physiological functions in inflammation and proliferation but can also trigger apoptosis through activation of IRAK and MyD88 [14], [15], [16]. While the microenvironment could benefit from the angiogenetic and the proliferative properties of IL1, the decoy-receptor might protect pancreatic cancers from apoptosis induced by the immune response [17].

The second major focus of dysregulations was found on the level of post-mitochondrial regulatory proteins, the inhibitors of apoptosis proteins (IAPs). This group of proteins inhibits the function of caspases and the apoptosome and thereby interferes both with the extrinsic and the intrinsic pathway. The IAPs are already known for their important role in carcinogenesis of other tumor entities and also PDAC [18], [19]. In our study, we found an upregulation of Survivin/BIRC5 and Livin/BIRC7 in microdissected tumor-tissue and we could validate the dysregulation of Livin/BIRC7 by quantitative RT-PCR and IHC.

The dysregulations in the group of IAPs might have a high clinical relevance, because the intrinsic pathway normally mediates the cytotoxic effect of irradiation and many chemotherapeutics [20], [21].

The computational analysis of the apoptosis pathway in PDAC thereby rendered a good accordance of our results with previous experimental references on apoptosis in pancreatic cancer. Using existing raw data from high-throughput technologies, we could partly reproduce experimental data. This data was put in the context of the complex intracellular apoptosis signaling by computational interaction analysis. Although a great amount of information can be assessed fast and descriptive by our approach, it is economically challenging to experimentally prove the findings. This must be considered a major disadvantage of our approach.

In Conclusion, the present study shows that by computational evaluation of data from gene expression analysis and public databases an acceptable virtual image of the apoptosis pathway might be given. Comparison of our data to previous publications rendered good accordance. By this approach we could identify defects at the level of cell death receptors and the inhibitor of apoptosis proteins, which might underlie the phenotype of distinct apoptosis resistance in PDAC. We could further for the first time identify IL1R2 as possible candidate gene.

## Materials and Methods

### Interaction prediction of the apoptosis pathway members

The apoptosis pathway related genes were assembled from electronic databases, such as the *Kyoto Encyclopedia of Genes and Genomes* (www.genome.ad.jp/kegg), *Gene Data Base of the National Center for Biotechnology Information* (www.ncbi.nlm.nih.gov) and *GeneMAPP* (www.genmapp.org). Keywords for the search were “apoptosis”, “cell death”, “cell death pathway”, “cell death receptor” (see **File S1**).

To evaluate interactions of the apoptosis associated proteins we initially queried databases with known protein-protein interactions such as NetPro (www.molecularconnections.com), SCOPPI (www.scoppi.org) and HPRD (www.hprd.org).

To find novel interactions we used two different methods. First, we used the structure-based prediction of protein interactions (see **File S2**). Most of the 103 apoptosis-associated genes were of unknown structure. We initially used the Genomic Threading Database (GTD) as a fold recognition method to assign structural families to the gene products [22]. Domains of proteins were then defined by the Structural Classification of Proteins, SCOP. Two domains are considered interacting if there are at least 5 residue pairs within 5 Å, in accord to the interface definitions [23]. Only domain-assignments with certain and high confidence by GTD were considered. To predict potential interactions of two given domains we then used SCOPPI [24]. This database provided evident domain-domain interactions, which served as structural templates for our original assigned domains. Two proteins are considered interacting if each contains a domain where there is a structural evidence for such a domain-domain interaction according to SCOPPI. The potential interactions were evaluated by analysis of the interface conservation. Information of the residues in the interface was again obtained from the SCOPPI database. The original protein sequence was aligned against the SCOPPI template sequence, a conservation of more than 30% of the interface residues was assumed to be sufficient to share the same interaction partner.

Second, we used a sequence-based prediction of protein interactions (see **File S2**). Therefore, we used NetPro, an expert curated and annotated database containing around 100,000 protein-protein interactions, for the prediction of an interaction of our proteins in question. Using this orthologous information and BLAST we searched for homologous interactions (>80% sequence identity) for a given protein pair. We only provide new interactions which were not confirmed before with NetPro or HPRD [25], [26]. To construct our pathway map, we set the cell death receptors as starting points of the signaling cascade. Interacting proteins were defined as downstream signaling proteins. Proteins which are known cell death receptor ligands were displayed as extra-cellular proteins.

### Gene expression analysis

For the construction of the virtual subarray data sets E-MEXP-950 and E-MEXP-1121 was used [27], [28].

Affymetrix probe set identifiers were obtained from Ensembl resulting in 189 probeset identifiers for 93 genes. For 10 genes no identifier could be obtained (see **File S1**). The Cel Files obtained from the Affymetrix MAS 5.0 software were used for further analysis. The Cel Files were loaded into dChip2006 (http://www.dchip.org), then normalized, and expression values were calculated using the PM/MM model. The expression values of the 189 probesets were exported and further explored using SAM (http://www-stat.stanford.edu/~tibs/sam/) and Excel (Microsoft, Redmond, WA). We scored genes as differentially expressed if they met the following criteria: a fold change >2 and a q value <5%. Heat maps were generated using dChip.

### Reverse Transcription Polymerase Reaction (RT-PCR)

1 ng of cDNA was used for a TaqMan assay (Applied Biosystems, Weiterstadt, Germany). The genes were amplified with a TaqMan Universal PCR Master Mix according to the manufacturer's instructions, with an ABI PRISM 5700 Sequence Detection System using gene specific primer and probes. Gene expression was quantified by the comparative cT-Method, normalizing cT-values to a housekeeping gene (β-actin) and calculating the relative expression values using the following primers: RT-PCR: BIRC7/Livin: ACT GAC CAG CCC TGA TTC C and CTC CAG GGA AAA CCC ACT TT; Actin: AAG CCA CCC CAC TTC TCT CTA A and AAT GCT ATC ACC TCC CCT GTG T; IL1R2: ATC AGC TTC TCT GGG GTC AA and GGT AGG CGC TCT CTA TGT GG [29].

### Immunohistochemistry

For immunohistochemistry, a tissue microarray (TMA) containing 16 PDAC samples was constructed. Of this TMA 5 µm sections were prepared using silanized slides (Menzel Gläser, Braunschweig, Germany). Immunohistochemistry for Livin/BIRC7 was performed using the streptavidin-biotin-peroxidase method as described previously and antigen retrieval was carried out in a microwave oven (250 W for 30 min in a citrate solution pH 6.0) [30], [31]. The primary antibody used was a mouse monoclonal antibody against the Livin/BIRC7 protein (#40958, Active Motif, Rixensart, Belgium). Normal colon mucosa and colorectal carcinoma were used as a positive control. As a negative control specimens were incubated without the primary antibody. Afterwards the slides were briefly counterstained with hematoxylin. Stained and unstained PDAC cells or ductal cells were counted and the ratio was generated. The staining intensity was evaluated semi-quantitatively by one pathologist (A.H.) without knowledge of the histopathologic and molecular data in 3 grades (negative, moderate and strong).

### Statistical analysis

For statistical analysis the t test and the chi square-test of “SPSS 13.0” for Windows were used.

### Literature search

To better assess changes in gene expression seen in our data, we conducted a comprehensive literature search on molecular defects of apoptosis in pancreatic cancer. Keywords were the name of the gene or protein together with the term “pancreatic carcinoma”, “pancreatic cancer”, “pancreas cancer” or “pancreatic ductal adenocarcinoma”. Included were studies on the level of the genome, gene expression and protein/functional studies on tumor tissue and/or cell lines. The literature search comprised publications until November 2009. See also **File S1**.

## Supporting Information

File S1 Data from our comprehensive literature search for the role of apoptosis-associated genes in pancreatic cancer. The table displays all genes, which were considered in our study. Please note that for most of the studies on the level of DNA, which means mutational studies, no quantitative statement concerning expression was made. Included were studies on the level of the genome, gene expression and protein/functional studies on tumor tissue and/or cell lines. The literature search comprised publications until December 2009. (--/- = less/slightly less expression than in normal tissue; +/- = expression depending on sample/cell-line, no general statement possible; 0 = no difference of expression to normal tissue/normal function of protein in experimental studies; ++/+ = higher/slightly higher expression than in normal tissue; ? = no quantitative statement in this study.)
(1.49 MB DOC)

File S2 Characteristics of our protein interaction prediction (A). Examples of structural models of three possible new interactions in the cell death pathway (more than 30% sequence and interface identity). The structural alignment between template and interacting protein structures is <2 Angstrom. 1 = Arts-Apollon; 2 = p16-ERK; 3 = p16-JNK (B).
(2.16 MB PPT)

File S3 Protein interactions from our database search and our interaction prediction analysis. Sheet one shows already known interactions from our database search. Sheet two shows putative interactions, proposed by our interaction prediction model.
(0.09 MB XLS)
