# Supplementary material for: Examination of Apoptosis Signaling in Pancreatic Cancer by Computational Signal Transduction Analysis
Source: PLoS One. 2010 Aug 19;5(8):e12243. doi: 10.1371/journal.pone.0012243 (PMC2924379; doi:10.1371/journal.pone.0012243)
Supplement: File S1 — Data from our comprehensive literature search for the role of apoptosis-associated genes in pancreatic cancer. The table displays all genes, which were considered in our study. Please note that for most of the studies on the level of DNA, which means mutational studies, no quantitative statement concerning expression was made. Included were studies on the level of the genome, gene expression and protein/functional studies on tumor tissue and/or cell lines. The literature search comprised publications until December 2009. (--/- = less/slightly less expression than in normal tissue; +/- = expression depending on sample/cell-line, no general statement possible; 0 = no difference of expression to normal tissue/normal function of protein in experimental studies; ++/+ = higher/slightly higher expression than in normal tissue; ? = no quantitative statement in this study.) (1.49 MB DOC) [file pone.0012243.s001.doc]

**Supporting Information 1: Members of the apoptosis pathway**

| **Ligands and cell death receptors** | | | | | | | | | | | | |
| --- | --- | --- | --- | --- | --- | --- | --- | --- | --- | --- | --- | --- |
|  | **Name** | **Gene Symbol** | **Affymetrix ID** | **RefSeq ID** | **Cell-line** | **Native Tumor** | **Level** | | | **iRNA** | **Expression** | **Reference** |
|  |  |  |  |  |  |  | **DNA** | **mRNA** | **Protein** |  |  |  |
| 1. **1** | FASL, CD95L | FASLG | 210865_at, 211333_s_at | NM_000639 | x |  |  | x | x |  | -- | [1] |
|  |  |  |  |  |  | x |  |  | x |  | 0 | [2] |
|  |  |  |  |  | x | x |  | x | x |  | ++ | [3] |
|  |  |  |  |  |  | x |  |  | x |  | ++ | [4] |
|  |  |  |  |  |  | x |  |  | x |  | ++ | [5] |
|  |  |  |  |  | x |  |  | x | x |  | ++ | [6] |
| 1. **2** | TRAIL | TNFSF10 | 202687_s_at, 202688_at, 214329_x_at | NM_003810 | x |  |  | x | x |  | 0 | [1] |
|  |  |  |  |  | x | x |  | x |  |  | ++ | [7] |
|  |  |  |  |  | x |  |  |  | x | x | + | [8] |
|  |  |  |  |  |  | x |  |  | x |  | 0 | [9] |
| 1. **3** | Fas, CD95, APO-1 | FAS | 204780_s_at, 204781_s_at, 215719_x_at, 216252_x_at, 233820_at | NM_000043 | x |  |  | x | x |  | 0 | [1] |
|  |  |  |  |  |  | x |  | x |  |  | 0 | [10] |
|  |  |  |  |  | x |  |  |  | x |  | - | [11] |
|  |  |  |  |  |  | x |  |  | x |  | -- | [2] |
|  |  |  |  |  | x | x |  | x | x |  | 0 | [3] |
|  |  |  |  |  |  | x |  |  | x |  | - | [5] |
|  |  |  |  |  | x |  |  | x | x |  | - | [12] |
|  |  |  |  |  | x |  |  | x | x |  | ++ | [6] |
|  |  |  |  |  | x |  |  |  | x |  | 0 | [13] |
|  |  |  |  |  | x |  |  |  | x |  | 0 | [14] |
| 1. **4** | TNFR1, CD120a | TNFRSF1A | 207643_s_at | NM_001065 | x |  |  |  | x |  | -/+ | [15] |
|  |  |  |  |  | x |  |  |  | x |  | 0 | [16] |
| 1. **5** | TNFR2, CD120b | TNFRSF1B | 203508_at | NM_001066 |  |  |  |  |  |  |  |  |
| 1. **6** | TRAILR-1, DR4, APO-2 | TNFRSF10A | 231775_at, 241371_at | NM_003844 | x |  |  | x | x |  | 0 | [1] |
|  |  |  |  |  |  | x |  | x |  |  | 0 | [10] |
|  |  |  |  |  | x |  |  | x | x |  | 0 | [17] |
|  |  |  |  |  | x |  |  |  | x |  | 0 | [18] |
|  |  |  |  |  | x | x |  | x |  |  | ++ | [7] |
|  |  |  |  |  |  | x |  |  | x |  | +/- | [19] |
|  |  |  |  |  | x |  |  | x | x |  | +/- | [20] |
|  |  |  |  |  | x |  |  |  | x |  | 0 | [21] |
|  |  |  |  |  | x |  |  |  | x |  | 0 | [14] |
|  |  |  |  |  |  | x |  |  | x |  | ++ | [9] |
| 1. **7** | TRAIL-R2, DR5, KILLER | TNFRSF10B | 209294_x_at, 209295_at, 210405_x_at | NM_003842 |  | x |  | x |  |  | 0 | [10] |
|  |  |  |  |  | x |  |  | x | x |  | 0 | [17] |
|  |  |  |  |  | x |  |  |  | x |  | 0 | [22] |
|  |  |  |  |  | x |  |  | x | x |  | 0 | [23] |
|  |  |  |  |  | x | x |  | x |  |  | ++ | [7] |
|  |  |  |  |  |  | x |  |  | x |  | +/- | [19] |
|  |  |  |  |  | x |  |  | x | x |  | +/- | [20] |
|  |  |  |  |  | x |  |  |  | x |  | + | [24] |
|  |  |  |  |  | x |  |  |  | x |  | 0 | [21] |
|  |  |  |  |  | x |  |  |  | x |  | 0 | [14] |
|  |  |  |  |  |  | x |  |  | x |  | 0 | [9] |
| 1. **8** | TRAIL-Rezeptor 3, DcR1 | TNFRSF10C | 211163_s_at, 206222_at, 210484_s_at, 234644_x_at | NM_003841 |  | x |  | x |  |  | 0 | [10] |
|  |  |  |  |  | x |  |  |  | x |  | 0 | [22] |
|  |  |  |  |  | x |  |  | x | x |  | -- | [17] |
|  |  |  |  |  | x |  |  | x | x |  | -- | [23] |
|  |  |  |  |  | x |  |  |  | x |  | -- | [15] |
|  |  |  |  |  | x |  |  | x | x |  | -- | [20] |
|  |  |  |  |  |  | x |  | x | x |  | 0 | [25] |
|  |  |  |  |  |  | x |  | x |  |  | 0 | [9] |
| 1. **9** | TRAIL-Rezeptor 4, DcR2, TRUNDD | TNFRSF10D | 210654_at, 227345_at | NM_003840 |  | x |  | x |  |  | 0 | [10] |
|  |  |  |  |  | x |  |  | x | x |  | 0 | [17] |
|  |  |  |  |  | x |  |  |  | x |  | 0 | [22] |
|  |  |  |  |  | x |  |  |  | x |  | ++ | [15] |
|  |  |  |  |  |  | x |  | x | x |  | ++ | [25] |
|  |  |  |  |  | x |  |  | x | x |  | -- | [20] |
|  |  |  |  |  |  | x |  | x |  |  | ++ | [9] |
| 1. **11** | DcR3 | TNFRSF6B | 206467_x_at, 211526_s_at, 216325_x_at | NM_032945 | x |  |  | x | x |  | ++ | [26] |
|  |  |  |  |  | x | x |  | x |  |  | + | [3] |
|  |  |  |  |  | x |  |  | x | x |  | + | [23] |
|  |  |  |  |  | x |  |  |  | x |  | ++ | [15] |
|  |  |  |  |  | x |  |  | x | x | x | ++ | [27] |
|  |  |  |  |  | x |  |  |  | x |  | ++ | [28] |
| 1. **10** | Osteoprotegrin | TNFRSF11B | 204932_at, 204933_s_at | NM_002546 |  | x |  | x |  |  | ++ | [10] |
| 1. **12** | IL1-R1 | IL1R1 | 215561_s_at | NM_000877.2 | x |  |  |  | x |  | ++ | [29] |
| 1. **13** | IL1-R2, IL1RB | IL1R2 | 205403_at, 211372_s_at | NM_004633 |  |  |  |  |  |  |  |  |
| 1. **14** | DR3, TRAMP, APO-3 | TNFRSF25 | 211282_x_at, 210847_x_at, 211841_s_at , 219423_x_at | NM_148965 | x |  |  | x | x |  | 0 | [1] |
|  |  |  |  |  | x |  |  |  | x | x | + | [30] |

| **Adaptor proteins** | | | | | | | | | | | | |
| --- | --- | --- | --- | --- | --- | --- | --- | --- | --- | --- | --- | --- |
|  | **Name** | **Gene Symbol** | **Affymetrix ID** | **RefSeq ID** | **Cell-line** | **Native Tumor** | **Level** | | | **iRNA** | **Expression** | **Reference** |
|  |  |  |  |  |  |  | **DNA** | **mRNA** | **Protein** |  |  |  |
| 1. **15** | Caspase 8, MACH, FLICE | CASP8 | 207686_s_at, 213373_s_at | NM_033358 | x |  |  | x | x |  | 0 | [17] |
|  |  |  |  |  | x | x |  | x |  |  | 0 | [3] |
|  |  |  |  |  | x |  |  | x |  |  | 0 | [31] |
|  |  |  |  |  | x |  |  |  | x |  | 0 | [14] |
|  |  |  |  |  | x |  |  |  | x | x | 0 | [32] |
| 1. **17** | FADD, MORT1 | FADD | 202535_at | NM_003824 | x |  |  | x | x |  | 0 | [17] |
|  |  |  |  |  | x |  |  | x |  |  | 0 | [3] |
|  |  |  |  |  | x |  |  |  | x |  | - | [18, 22] |
|  |  |  |  |  | x |  |  |  | x |  | 0 | [23] |
|  |  |  |  |  | x |  |  | x |  |  | - | [12] |
|  |  |  |  |  | x |  |  |  | x |  | 0 | [21] |
|  |  |  |  |  | x |  |  |  | x |  | 0 | [14] |
|  |  |  |  |  | x |  |  |  | x | x | 0 | [32] |
| 1. **16** | FLIP, c-FLIP | CFLAR | 208485_x_at, 209508_x_at, 210563_x_at, 210564_x_at, 211316_x_at, 211317_s_at, 211862_x_at, 214486_x_at | NM_003879 | x |  |  |  | x |  | 0 | [33] |
|  |  |  |  |  | x |  |  | x | x |  | ++ | [17] |
|  |  |  |  |  | x | x |  | x |  |  | ++ | [3] |
|  |  |  |  |  | x |  |  |  | x |  | ++ | [22] |
|  |  |  |  |  | x |  |  |  | x |  | 0 | [15] |
|  |  |  |  |  |  | x |  | x |  |  | ++ | [34] |
|  |  |  |  |  | x |  |  |  | x | x | ++ | [35] |
|  |  |  |  |  | x |  |  |  | x | x | ++ | [21] |
|  |  |  |  |  | x |  |  |  | x |  | ++ | [8] |
| 1. **18** | TRADD | TRADD | 1729_at, 205641_s_at | NM_003789 |  | x |  | x |  |  | ++ | [34] |
| 1. **19** | RIP | RIP | 228183_s_at, 216962_at | NM_001033002 | x |  |  |  | x |  | 0 | [14] |
|  |  |  |  |  | x |  |  |  | x | x | + | [21] |
|  |  |  |  |  | x |  |  |  | x |  | 0 | [14] |
| 1. **20** | FAP-1, PTP-BAS, PTPL1 | PTPN13 | 204201_s_at | NM_080683 | x | x |  | x | x |  | ++ | [3] |
|  |  |  |  |  |  | x |  |  | x |  | ++ | [36] |
|  |  |  |  |  | x |  |  |  | x |  | -- | [15] |
| 1. **21** | DENN, IG20 | MADD | 210252_s_at, 38398_at | NM_130470 |  |  |  |  |  |  |  |  |
| 1. **22** | RAIDD | CRADD | no probe-set identified | NM_003805 |  |  |  |  |  |  |  |  |
| 1. **23** | TRAF 6 | TRAF6 | 205558_at | NM_145803 |  |  |  |  |  |  |  |  |
| 1. **24** | TRAF 2 | TRAF2 | 204413_at | NM_021138 | x |  | x |  | x |  | ++ | [37] |
|  |  |  |  |  | x |  |  |  | x |  | 0 | [14] |
| 1. **25** | TRAF 1 | TRAF1 | 205599_at, 235116_at | NM_005658 |  |  |  |  |  |  |  |  |
| 1. **26** | MyD88 | MYD88 | 209124_at | NM_002468 | x |  |  |  | x | x | + | [38] |
| 1. **27** | IRAK, pelle | IRAK1 | 201587_s_at | NM_001569 |  | x |  | x |  |  | ++ | [34] |
| 1. **28** | IRAK, pelle | IRAK2 | 220034_at | NM_001570 |  |  |  |  |  |  |  |  |
| 1. **29** | IRAK, pelle | IRAK3 | 220033_at | NM_007199 |  |  |  |  |  |  |  |  |
| 1. **30** | IRAK, pelle | IRAK4 | 219618_at | NM_016123 |  |  |  |  |  |  |  |  |
| 1. **31** | DAXX | DAXX | 201763_s_at, 216038_x_at | NM_001350 |  |  |  |  |  |  |  |  |

| **Intracellular proteins** | | | | | | | | | | | | |
| --- | --- | --- | --- | --- | --- | --- | --- | --- | --- | --- | --- | --- |
|  | **Name** | **Gene Symbol** | **Affymetrix ID** | **RefSeq ID** | **Cell-line** | **Native tumor** | **Level** | | | **iRNA** | **Expression** | **Reference** |
|  |  |  |  |  |  |  | **DNA** | **mRNA** | **Protein** |  |  |  |
| 1. **32** | NAIP (baculoviral IAP repeat-containing 1) | BIRC1 | 204860_s_at, 204861_s_at | NM_004536 | x |  |  | x |  |  | 0 | [39] |
| 1. **33** | cIAP1, Hiap-2 | BIRC2 | 202076_at | NM_001166 | x |  |  |  | x |  | ++ | [15] |
|  |  |  |  |  | x |  | x | x |  |  | ? | [40] |
|  |  |  |  |  | x |  |  | x |  |  | + | [39] |
|  |  |  |  |  | x |  |  |  | x |  | + | [41] |
|  |  |  |  |  | x | x |  | x | x |  | 0 | [42] |
| 1. **34** | cIAP2, Hiap-1 | BIRC3 | 210538_s_at, 230499_at | NM_001165 | x |  | x | x |  |  | ? | [40] |
|  |  |  |  |  |  | x |  | x |  |  | ++ | [34] |
|  |  |  |  |  | x |  |  |  | x |  | -- | [15] |
|  |  |  |  |  | x |  |  | x | x |  | ++ | [20] |
|  |  |  |  |  | x | x |  | x | x | x | + | [39] |
|  |  |  |  |  | x |  |  |  | x |  | + | [41] |
|  |  |  |  |  | x | x |  | x | x |  | ++ | [42] |
| 1. **35** | XIAP | BIRC4 | 206536_s_at, 206537_at, 225858_s_at, 225859_at, 228363_at, 243026_x_at, 235222_x_at | NM_001167 | x |  |  | x | x |  | 0 | [17] |
|  |  |  |  |  | x |  |  |  | x |  | + | [43] |
|  |  |  |  |  | x |  |  | x | x |  | ++ | [20] |
|  |  |  |  |  | x | x |  | x | x | x | +/0 | [39] |
|  |  |  |  |  | x |  |  |  | x | x | + | [41] |
|  |  |  |  |  | x |  |  |  | x |  | + | [17] |
|  |  |  |  |  | x | x |  | x | x | x | ++ | [44] |
|  |  |  |  |  |  | x |  |  | x |  | ++ | [45] |
|  |  |  |  |  | x |  |  |  | x |  | ++ | [46] |
| 1. **36** | Survivin | BIRC5 | 202094_at, 202095_s_at, 210334_x_at | NM_001168 |  | x |  |  | x |  | 0 | [47] |
|  |  |  |  |  |  | x |  |  | x |  | ++ | [48] |
|  |  |  |  |  |  | x |  |  | x |  | + | [49] |
|  |  |  |  |  | x | x |  | x | x |  | ++ | [50] |
|  |  |  |  |  |  | x |  |  | x |  | ++ | [51] |
|  |  |  |  |  | x |  |  |  | x |  | ++ | [15] |
|  |  |  |  |  | x |  |  | x | x |  | ++ | [52] |
|  |  |  |  |  | x |  |  | x | x |  | ++ | [20] |
|  |  |  |  |  | x | x |  | x | x |  | ++ | [39] |
|  |  |  |  |  | x |  |  |  | x |  | + | [41] |
| 1. **37** | Apollon | BIRC6 | no probe-set identified | NM_016252 | x |  |  | x |  |  | 0 | [39] |
| 1. **38** | LIVIN, ML-IAP | BIRC7 | 220451_s_at | NM_139317 | x | x |  | x | x |  | ++ | [39] |
|  |  |  |  |  | x |  |  |  | x |  | - | [41] |
| 1. **39** | ILP-2 | BIRC8 | no probe-set identified | NM_033341 |  |  |  |  |  |  |  |  |
| 1. **40** | PIDD | LRDD | 219019_at, 221640_s_at | NM_018494 |  |  |  |  |  |  |  |  |
| 1. **41** | DFFA | DFFA | 203277_at, 226116_at | NM_004401 |  |  |  |  |  |  |  |  |
| 1. **42** | Caspase 3 | CASP3 | 202763_at | NM_004346 | x |  |  | x | x |  | - | [17] |
|  |  |  |  |  | x |  |  | x | x |  | 0 | [31] |
|  |  |  |  |  | x |  |  |  | x |  | ? | [23] |
|  |  |  |  |  | x |  |  |  | x |  | 0 | [15] |
|  |  |  |  |  | x |  |  |  | x |  | 0 | [53] |
|  |  |  |  |  | x |  |  |  | x |  | 0 | [14] |
| 1. **43** | Caspase 6 | CASP6 | 209790_s_at, 211464_x_at | NM_001226 | x |  |  | x |  |  | 0 | [31] |
| 1. **44** | Caspase 7 | CASP7 | 207181_s_at | NM_001227 | x |  |  | x |  |  | 0 | [31] |
|  |  |  |  |  | x |  |  |  | x |  | 0 | [53] |
| 1. **45** | Caspase 9 | CASP9 | 203984_s_at, 210775_x_at, 240437_at | NM_001229 | x |  |  | x | x |  | 0 | [17] |
|  |  |  |  |  | x |  |  | x |  |  | 0 | [31] |
|  |  |  |  |  | x |  |  |  | x |  | 0 | [15] |
|  |  |  |  |  | x |  |  |  | x |  | 0 | [53] |
| 1. **46** | Caspase 10, FLICE-2 | CASP10 | 205467_at, 210708_x_at, 211888_x_at | NM_001230 | x |  |  |  | x |  | 0 | [53] |
| 1. **48** | Calpain | CAPN2 | 208683_at | NM_001748 |  |  |  |  |  |  |  |  |
| 1. **49** | Calpain | CAPNS1 | 200001_at | NM_001003962 |  |  |  |  |  |  |  |  |
| 1. **50** | Calmodulin 2 | CALM2 | no probe-set identified | NM_001743 |  |  |  |  |  |  |  |  |
| 1. **51** | Calmodulin 1 | CALM1 | 200622_x_at, 200623_s_at, 200653_s_at, 200655_s_at, 207243_s_at, 211984_at, 211985_s_at, 213688_at | NM_006888 |  |  |  |  |  |  |  |  |
|  | Hsp70 | HSPA1A | no probe-set identified | NM_005345 | x |  |  | x | x |  | ++ | [54] |
|  |  |  |  |  | x | x |  | x | x | x | ++ | [55] |
|  |  |  |  |  | x |  |  | x | x | x | ++ | [56] |
| 1. **52** | p16INK4 | CDKN2A | 207039_at, 209644_x_at | NM_000077 |  | x | x | x | x |  | -- | [57] |
|  |  |  |  |  | x |  | x |  |  |  | -- | [58] |

| **Mtchondrial proteins** | | | | | | | | | | | | |
| --- | --- | --- | --- | --- | --- | --- | --- | --- | --- | --- | --- | --- |
|  | **Name** | **Gene Symbol** | **Affymetrix ID** | **RefSeq ID** | **Cell-line** | **Native Tumor** | **Level** | | | **iRNA** | **Expression** | **Reference** |
|  |  |  |  |  |  |  | **DNA** | **mRNA** | **Protein** |  |  |  |
| 1. **79** | Bid | BID | 204493_at, 211725_s_at, 227143_s_at | NM_197966 | x |  |  |  | x |  | 0 | [59] |
|  |  |  |  |  | x |  |  |  | x |  | -- | [22] |
|  |  |  |  |  | x |  |  |  | x |  | 0 | [19] |
|  |  |  |  |  | x |  |  |  | x |  | 0 | [14] |
|  |  |  |  |  | x |  |  |  | x | x | 0 | [60] |
| 1. **73** | BFL1, BCL2-related protein A1 | BCL2A1 | 205681_at | NM_004049 | x |  |  |  | x |  | 0 | [61] |
| 1. **80** | Humanin | HN1 | 222396_at | NM_001002032 |  |  |  |  |  |  |  |  |
|  | Mule | HUWE1 | no probe-set identified | NM_031407 |  |  |  |  |  |  |  |  |
| 1. **74** | BAG-3 | BAG3 | 217911_s_at | NM_004281 |  | x |  | x | x |  | ++ | [62] |
| 1. **54** | Mcl-1 | MCL1 | 200796_s_at, 200797_s_at, 200798_x_at, 241722_x_at | NM_021960 | x | x |  |  | x |  | ++ | [63] |
|  |  |  |  |  |  | x |  | x |  |  | + | [64] |
|  |  |  |  |  | x |  |  |  | x |  | 0 | [65] |
|  |  |  |  |  | x |  |  |  | x | x | + | [66] |
|  |  |  |  |  | x |  |  |  | x | x | ++ | [67] |
| 1. **61** | Bcl-xL | BCL2L1 | no probe-set identified | NM_138578 | x |  |  | x | x |  | 0 | [17] |
|  |  |  |  |  |  | x |  |  | x |  | +/- | [68] |
|  |  |  |  |  |  | x |  | x | x |  | ++ | [69] |
|  |  |  |  |  | x |  |  |  | x |  | 0 | [70] |
|  |  |  |  |  | x |  |  |  | x |  | ++ | [22] |
|  |  |  |  |  | x | x |  | x | x |  | ++ | [63] |
|  |  |  |  |  | x |  |  |  | x |  | ++ | [15] |
|  |  |  |  |  | x |  |  |  | x | x | ++ | [71] |
|  |  |  |  |  | x |  |  |  | x |  | 0 | [65] |
| 1. **58** | Bcl-2 | BCL2 | 207005_s_at, 203685_at, 203684_s_at | NM_000633 |  | x |  |  | x |  | 0 | [68] |
|  |  |  |  |  |  | x |  | x | x |  | ++ | [69] |
|  |  |  |  |  | x |  |  |  | x |  | 0 | [70] |
|  |  |  |  |  |  | x |  |  | x |  | ++ | [48] |
|  |  |  |  |  | x |  |  |  | x |  | 0 | [15] |
|  |  |  |  |  | x | x |  |  | x |  | - | [63] |
|  |  |  |  |  | x |  |  | x |  |  | +/- | [64] |
|  |  |  |  |  | x |  |  | x | x | x | ++ | [72] |
|  |  |  |  |  | x |  |  |  | x |  | 0 | [65] |
| 1. **66** | Bcl-w | BCL2L2 | 209311_at | NM_020396 |  |  |  |  |  |  |  |  |
| 1. **72** | Bcl-B, DIVA | BCL2L10 | 221320_at, 236491_at | NM_020396 |  |  |  |  |  |  |  |  |
| 1. **55** | BAX | BAX | 211833_s_at | NM_138761 | x |  |  | x | x |  | 0 | [17] |
|  |  |  |  |  |  | x |  |  | x |  | +/- | [68] |
|  |  |  |  |  |  | x |  | x | x |  | ++ | [69] |
|  |  |  |  |  | x |  |  |  | x |  | 0 | [70] |
|  |  |  |  |  |  | x |  | x | x |  | ++ | [73] |
|  |  |  |  |  | x |  |  | x |  |  | -/+ | [64] |
|  |  |  |  |  | x |  |  | x | x |  | 0 | [72] |
|  |  |  |  |  | x |  |  |  | x |  | 0 | [65] |
|  |  |  |  |  | x |  |  |  | x | x | 0 | [60] |
| 1. **56** | BAK 1 | BAK1 | 203728_at | NM_001188 |  | x |  |  | x |  | -/+ | [68] |
|  |  |  |  |  | x |  |  | x |  |  | -/+ | [64] |
|  |  |  |  |  | x |  |  |  | x | x | 0 | [65] |
|  |  |  |  |  | x |  |  |  | x | x | 0 | [60] |
| 1. **57** | Bim/Bod | BCL2L11 | 208536_s_at, 222343_at, 225606_at | NM_006538 | x |  |  |  | x |  | 0 | [74] |
|  |  |  |  |  | x |  |  |  | x | x | + | [75] |
| 1. **59** | PUMA, JFY1 | BBC3 | 211692_s_at | NM_014417 |  |  |  |  |  |  |  |  |
|  |  |  |  |  | x |  |  |  | x | x | + | [75] |
| 1. **60** | NOXA | PMAIP1 | 204285_s_at, 204286_s_at | NM_021127 | x |  |  | x |  |  | + | [76] |
| 1. **62** | Bcl-X_S_ | BCL2L1 | 206665_s_at, 215037_s_at, 212312_at | NM_001191 | x |  |  |  | x |  | 0 | [70] |
|  |  |  |  |  | x |  |  | x |  |  | -/+ | [64] |
| 1. **65** | Bad | BBC2, BAD | 1861_at, 209364_at | NM_004322 | x |  |  |  | x |  | 0 | [65] |
| 1. **67** | BNIP 3 | BNIP3 | 201848_s_at, 201849_at | NM_004052 | x |  |  | x | x |  | 0 | [77] |
|  |  |  |  |  | x | x |  | x | x |  | -- | [78] |
|  |  |  |  |  | x | x |  | x | x |  | -- | [79] |
|  |  |  |  |  |  | x |  | x | x |  | -- | [80] |
|  |  |  |  |  | x | x |  | x |  |  | -- | [81] |
|  |  |  |  |  | x | x |  | x | x |  | -/0 | [82] |
| 1. **68** | BIK | BIK | no probe-set identified | NM_001197 |  |  |  |  |  |  |  |  |
| 1. **69** | BLK | BLK | 206255_at | NM_001715 |  | x |  | x |  |  | + | [64] |
| 1. **70** | harakiri | HRK | 206864_s_at, 206865_at, 237187_at | NM_003806 |  |  |  |  |  |  |  |  |
| 1. **71** | BMF | BMF | 226530_at | NM_001003940 |  |  |  |  |  |  |  |  |
| 1. **75** | endonuclease G | ENDOG | 204824_at | NM_004435 |  |  |  |  |  |  |  |  |
| 1. **77** | AMID | AMID | 224461_s_at, 228445_at | NM_032797 |  |  |  |  |  |  |  |  |
| 1. **78** | APAF 1/CED4 | APAF1 | 204859_s_at, 211553_x_at, 211554_s_at | NM_013229 | x |  |  | x |  |  | 0 | [31] |
| 1. **63** | HtrA2/Omi | HTRA2 | 203089_s_at, 211152_s_at | NM_013247 | x |  |  | x |  |  | 0 | [39] |
| 1. **76** | SMAC/DIABLO | DIABLO | 219350_s_at | NM_019887 | x |  |  | x | x |  | 0 | [17] |
|  |  |  |  |  | x |  |  | x | x |  | 0 | [20] |
|  |  |  |  |  | x |  |  | x |  |  | 0 | [39] |
|  |  |  |  |  | x |  |  |  | x |  | + | [41] |
|  |  |  |  |  | x |  |  |  | x |  | 0 | [60] |
| 1. **53** | BIT1 | PTRH2 | 218732_at | NM_001015509 |  |  |  |  |  |  |  |  |
| 1. **64** | AIF | PDCD8 | 205512_s_at | NM_004208 | x |  |  |  | x |  | 0 | [83] |
|  |  |  |  |  | x |  |  |  | x | x | 0 | [60] |
| 1. **47** | ARTS | SEPT4 | 210657_s_at | NM_004574 |  |  |  |  |  |  |  |  |
|  | Cytochrome C |  | no probe-set identified |  | x |  |  |  | x |  | 0 | [54] |
|  |  |  |  |  | x |  |  |  | x |  | 0 | [60] |

| **Modulating proteins** | | | | | | | | | | | | |
| --- | --- | --- | --- | --- | --- | --- | --- | --- | --- | --- | --- | --- |
|  | **Name** | **Gene Symbol** | **Affymetrix ID** | **RefSeq ID** | **Cell-line** | **Native Tumor** | **Level** | | | **iRNA** | **Expression** | **Reference** |
|  |  |  |  |  |  |  | **DNA** | **mRNA** | **Protein** |  |  |  |
| 1. **82** | NIK | MAP3K14 | 205192_at | NM_003954 |  |  |  |  |  |  |  |  |
| 1. **83** | IKBA | NFKBIA | 201502_s_at | NM_020529 | x |  |  |  | x |  | 0 | [14] |
|  |  |  |  |  | x |  |  |  | x |  | 0 | [27] |
| 1. **84** | IKK-beta, IKK1 | IKBKB | 209341_s_at, 209342_s_at, 211027_s_at | NM_001556 |  | x |  |  | x |  | 0 | [84] |
|  |  |  |  |  | x | x |  | x | x |  | ++ | [85] |
|  |  |  |  |  | x |  |  |  | x |  | 0 | [27] |
| 1. **85** | IKK-alpha, IKK2 | CHUK | 209666_s_at | NM_001278 |  | x |  |  | x |  | 0 | [84] |
|  |  |  |  |  | x |  |  |  | x |  | 0 | [27] |
| 1. **86** | NF-κB | NFKB1 | 209239_at | NM_003998 |  | x |  |  | x |  | 0 | [33] |
|  |  |  |  |  |  | x |  |  | x |  | ++ | [84] |
|  |  |  |  |  | x | x |  |  | x |  | ++ | [85] |
|  |  |  |  |  | x |  |  |  | x |  | ++ | [61, 86] |
|  |  |  |  |  | x |  |  |  | x | x | ++ | [87] |
|  |  |  |  |  | x |  |  |  | x | x | ++ | [88] |
| 1. **95** | K-ras | KRAS | 212983_at | NM_005343 | x | x |  |  | x |  | ++ | [89] |
|  |  |  |  |  |  | x | x |  |  |  | + | [90] |
|  |  |  |  |  |  | x | x |  |  |  | + | [91] |
|  |  |  |  |  | x |  | x |  |  |  | + | [58] |
| 1. **97** | JNK1 | MAPK8 | 210477_x_at, 210671_x_at, 226046_at, 226048_at, 229664_at | NM_002750 | x |  |  | x | x |  | 0 | [92] |
|  |  |  |  |  | x |  |  | x | x |  | ++ | [6] |
|  |  |  |  |  | x |  |  |  | x |  | 0 | [14] |
| 1. **98** | JNKK | MAP2K4 | no probe-set identified | NM_003010 |  |  |  |  |  |  |  |  |
| 1. **92** | AP-1 | JUN | 201464_x_at, 201465_s_at, 201466_s_at | NM_002228 |  | x |  |  | x |  | ++ | [93] |
| 1. **100** | c-fos | FOS | 209189_at | NM_005252 | x |  | x |  |  |  | + | [94] |
|  |  |  |  |  |  | x |  |  | x |  | ++ | [95] |
| 1. **90** | ERK 1 | MAPK3 | 212046_x_at | NM_002746 | x | x |  |  | x |  | + | [89] |
|  |  |  |  |  |  | x |  |  | x |  | ++ | [93] |
|  |  |  |  |  |  | x |  |  | x |  | ++ | [96] |
|  |  |  |  |  | x |  |  | x | x |  | ++ | [6] |
|  |  |  |  |  | x |  |  |  | x |  | 0 | [14] |
|  |  |  |  |  | x |  |  |  | x |  | ++ | [97] |
| 1. **91** | ERK 2 | MAPK1 | 208351_s_at, 212271_at, 224621_at | NM_002745 | x | x |  |  | x |  | + | [89] |
|  |  |  |  |  |  | x |  |  | x |  | ++ | [93] |
|  |  |  |  |  |  | x |  |  | x |  | ++ | [96] |
|  |  |  |  |  | x |  |  | x | x |  | ++ | [6] |
| 1. **96** | PEA-15 | PEA15 | 200787_s_at, 200788_s_at | NM_003768 |  |  |  |  |  |  |  |  |
| 1. **88** | C/EBP | CEBPG | 204203_at, 225527_at | NM_001806 |  |  |  |  |  |  |  |  |
| 1. **87** | ASK1 | MAP3K5 | 203836_s_at, 203837_at | NM_005923 |  |  |  |  |  |  |  |  |
| 1. **81** | Protein Kinase Cμ | Prkcm | 205880_at | NM_008858 |  | x |  |  | x |  | ++ | [98] |
|  |  |  |  |  | x |  |  |  | x |  | 0 | [99] |
| 1. **94** | NFAT2 | NFATC1 | 208196_x_at, 209664_x_at, 211105_s_at | NM_006162 |  |  |  |  |  |  |  |  |
| 1. **89** | UCP2 | UCP2 | 208997_s_at, 208998_at | NM_003355 |  |  |  |  |  |  |  |  |
| 1. **93** | Microtubule-associated Protein | MAP2 | 210015_s_at | NM_002374 |  | x |  |  | x |  | ++ | [96] |
| 1. **99** | p53 | TP53 | 201746_at, 211300_s_at | NM_000546 |  | x |  |  | x |  | ++ | [68] |
|  |  |  |  |  |  | x | x |  |  |  | ? | [90] |
|  |  |  |  |  |  | x | x |  |  |  | -- | [91] |
|  |  |  |  |  | x |  |  |  | x |  | - | [100] |
|  |  |  |  |  |  | x | x |  |  |  | -- | [58] |
|  |  |  |  |  |  | x |  |  | x |  | ++ | [101] |

**References for defects of the apoptosis pathway in pancreatic cancer**

1. Ringel B, Ibrahim SM, Kohler H, Ringel J, Koczan D, Liebe S, Lohr M, & Thiesen HJ: Apoptotic molecules in pancreatic carcinoma cell lines. Ann N Y Acad Sci 1999; 880:175-178.

2. Bernstorff WV, Glickman JN, Odze RD, Farraye FA, Joo HG, Goedegebuure PS, & Eberlein TJ: Fas (CD95/APO-1) and Fas ligand expression in normal pancreas and pancreatic tumors. Implications for immune privilege and immune escape. Cancer 2002; 94:2552-2560.

3. Elnemr A, Ohta T, Yachie A, Kayahara M, Kitagawa H, Fujimura T, Ninomiya I, Fushida S, Nishimura GI, Shimizu K, & Miwa K: Human pancreatic cancer cells disable function of Fas receptors at several levels in Fas signal transduction pathway. Int J Oncol 2001; 18:311-316.

4. Ohta T, Elnemr A, Kitagawa H, Kayahara M, Takamura H, Fujimura T, Nishimura G, Shimizu K, Yi SQ, & Miwa K: Fas ligand expression in human pancreatic cancer. Oncol Rep 2004; 12:749-754.

5. Pernick NL, Sarkar FH, Tabaczka P, Kotcher G, Frank J, & Adsay NV: Fas and Fas ligand expression in pancreatic adenocarcinoma. Pancreas 2002; 25:e36-41.

6. Kornmann M, Ishiwata T, Kleeff J, Beger HG, & Korc M: Fas and Fas-ligand expression in human pancreatic cancer. Ann Surg 2000; 231:368-379.

7. Ozawa F, Friess H, Kleeff J, Xu ZW, Zimmermann A, Sheikh MS, & Buchler MW: Effects and expression of TRAIL and its apoptosis-promoting receptors in human pancreatic cancer. Cancer Lett 2001; 163:71-81.

8. Murtaza I, Saleem M, Adhami VM, Hafeez BB, & Mukhtar H: Suppression of cFLIP by lupeol, a dietary triterpene, is sufficient to overcome resistance to TRAIL-mediated apoptosis in chemoresistant human pancreatic cancer cells. Cancer Res 2009; 69:1156-1165.

9. Sanlioglu AD, Dirice E, Elpek O, Korcum AF, Ozdogan M, Suleymanlar I, Balci MK, Griffith TS, & Sanlioglu S: High TRAIL death receptor 4 and decoy receptor 2 expression correlates with significant cell death in pancreatic ductal adenocarcinoma patients. Pancreas 2009; 38:154-160.

10. Satoh K, Kaneko K, Hirota M, Masamune A, Satoh A, & Shimosegawa T: Tumor necrosis factor-related apoptosis-inducing ligand and its receptor expression and the pathway of apoptosis in human pancreatic cancer. Pancreas 2001; 23:251-258.

11. Monti P, Marchesi F, Reni M, Mercalli A, Sordi V, Zerbi A, Balzano G, Di Carlo V, Allavena P, & Piemonti L: A comprehensive in vitro characterization of pancreatic ductal carcinoma cell line biological behavior and its correlation with the structural and genetic profile. Virchows Arch 2004; 445:236-247.

12. Radfar S, Davrinche C, & Hollande E: Serial in vivo loss and in vitro gain of Fas expression and function in human cancerous pancreatic duct cells. Int J Cancer 2005; 115:214-223.

13. Christgen M, Schniewind B, Jueschke A, Ungefroren H, & Kalthoff H: Gemcitabine-mediated apoptosis is associated with increased CD95 surface expression but is not inhibited by DN-FADD in Colo357 pancreatic cancer cells. Cancer Lett 2005; 227:193-200.

14. Siegmund D, Klose S, Zhou D, Baumann B, Roder C, Kalthoff H, Wajant H, & Trauzold A: Role of caspases in CD95L- and TRAIL-induced non-apoptotic signalling in pancreatic tumour cells. Cell Signal 2007; 19:1172-1184.

15. Bai J, Sui J, Demirjian A, Vollmer CM, Jr., Marasco W, & Callery MP: Predominant Bcl-XL knockdown disables antiapoptotic mechanisms: tumor necrosis factor-related apoptosis-inducing ligand-based triple chemotherapy overcomes chemoresistance in pancreatic cancer cells in vitro. Cancer Res 2005; 65:2344-2352.

16. Baran B, Bechyne I, Siedlar M, Szpak K, Mytar B, Sroka J, Laczna E, Madeja Z, Zembala M, & Czyz J: Blood monocytes stimulate migration of human pancreatic carcinoma cells in vitro: the role of tumour necrosis factor - alpha. Eur J Cell Biol 2009; 88:743-752.

17. Mori T, Doi R, Toyoda E, Koizumi M, Ito D, Kami K, Kida A, Masui T, Kawaguchi Y, & Fujimoto K: Regulation of the resistance to TRAIL-induced apoptosis as a new strategy for pancreatic cancer. Surgery 2005; 138:71-77.

18. Trauzold A, Schmiedel S, Oestern S, Christgen M, Westphal S, Roederm C, & Kalthoff H: Concerted deregulations of multiple apoptosis-controlling genes in pancreatic carcinoma cells. Ann N Y Acad Sci 2003; 1010:510-513.

19. Hylander BL, Pitoniak R, Penetrante RB, Gibbs JF, Oktay D, Cheng J, & Repasky EA: The anti-tumor effect of Apo2L/TRAIL on patient pancreatic adenocarcinomas grown as xenografts in SCID mice. J Transl Med 2005; 3:22.

20. Vogler M, Durr K, Jovanovic M, Debatin KM, & Fulda S: Regulation of TRAIL-induced apoptosis by XIAP in pancreatic carcinoma cells. Oncogene 2006;

21. Wang P, Zhang J, Bellail A, Jiang W, Hugh J, Kneteman NM, & Hao C: Inhibition of RIP and c-FLIP enhances TRAIL-induced apoptosis in pancreatic cancer cells. Cell Signal 2007; 19:2237-2246.

22. Trauzold A, Schmiedel S, Roder C, Tams C, Christgen M, Oestern S, Arlt A, Westphal S, Kapischke M, Ungefroren H, & Kalthoff H: Multiple and synergistic deregulations of apoptosis-controlling genes in pancreatic carcinoma cells. Br J Cancer 2003; 89:1714-1721.

23. Ibrahim SM, Ringel J, Schmidt C, Ringel B, Muller P, Koczan D, Thiesen HJ, & Lohr M: Pancreatic adenocarcinoma cell lines show variable susceptibility to TRAIL-mediated cell death. Pancreas 2001; 23:72-79.

24. DeRosier LC, Buchsbaum DJ, Oliver PG, Huang ZQ, Sellers JC, Grizzle WE, Wang W, Zhou T, Zinn KR, Long JW, & Vickers SM: Combination treatment with TRA-8 anti death receptor 5 antibody and CPT-11 induces tumor regression in an orthotopic model of pancreatic cancer. Clin Cancer Res 2007; 13:5535s-5543s.

25. Liao Q, Friess H, Kleeff J, & Buchler MW: Differential expression of TRAIL-R3 and TRAIL-R4 in human pancreatic cancer. Anticancer Res 2001; 21:3153-3159.

26. Tsuji S, Hosotani R, Yonehara S, Masui T, Tulachan SS, Nakajima S, Kobayashi H, Koizumi M, Toyoda E, Ito D, Kami K, Mori T, Fujimoto K, Doi R, & Imamura M: Endogenous decoy receptor 3 blocks the growth inhibition signals mediated by Fas ligand in human pancreatic adenocarcinoma. Int J Cancer 2003; 106:17-25.

27. Chen PH & Yang CR: Decoy receptor 3 expression in AsPC-1 human pancreatic adenocarcinoma cells via the phosphatidylinositol 3-kinase-, Akt-, and NF-kappa B-dependent pathway. J Immunol 2008; 181:8441-8449.

28. Yang CR, Guh JH, Teng CM, Chen CC, & Chen PH: Combined treatment with denbinobin and Fas ligand has a synergistic cytotoxic effect in human pancreatic adenocarcinoma BxPC-3 cells. Br J Pharmacol 2009; 157:1175-1185.

29. Sawai H, Funahashi H, Yamamoto M, Okada Y, Hayakawa T, Tanaka M, Takeyama H, & Manabe T: Interleukin-1alpha enhances integrin alpha(6)beta(1) expression and metastatic capability of human pancreatic cancer. Oncology 2003; 65:167-173.

30. Murtaza I, Adhami VM, Hafeez BB, Saleem M, & Mukhtar H: Fisetin, a natural flavonoid, targets chemoresistant human pancreatic cancer AsPC-1 cells through DR3-mediated inhibition of NF-kappaB. Int J Cancer 2009; 125:2465-2473.

31. Gerhard MC, Schmid RM, & Hacker G: Analysis of the cytochrome c-dependent apoptosis apparatus in cells from human pancreatic carcinoma. Br J Cancer 2002; 86:893-898.

32. Basu A & Haldar S: Combinatorial effect of epigallocatechin-3-gallate and TRAIL on pancreatic cancer cell death. Int J Oncol 2009; 34:281-286.

33. Thomas RP, Farrow BJ, Kim S, May MJ, Hellmich MR, & Evers BM: Selective targeting of the nuclear factor-kappaB pathway enhances tumor necrosis factor-related apoptosis-inducing ligand-mediated pancreatic cancer cell death. Surgery 2002; 132:127-134.

34. Grutzmann R, Saeger HD, Luttges J, Schackert HK, Kalthoff H, Kloppel G, & Pilarsky C: Microarray-based gene expression profiling in pancreatic ductal carcinoma: status quo and perspectives. Int J Colorectal Dis 2004; 19:401-413.

35. Mori T, Doi R, Kida A, Nagai K, Kami K, Ito D, Toyoda E, Kawaguchi Y, & Uemoto S: Effect of the XIAP inhibitor Embelin on TRAIL-induced apoptosis of pancreatic cancer cells. J Surg Res 2007; 142:281-286.

36. Ungefroren H, Kruse ML, Trauzold A, Roeschmann S, Roeder C, Arlt A, Henne-Bruns D, & Kalthoff H: FAP-1 in pancreatic cancer cells: functional and mechanistic studies on its inhibitory role in CD95-mediated apoptosis. J Cell Sci 2001; 114:2735-2746.

37. Trauzold A, Roder C, Sipos B, Karsten K, Arlt A, Jiang P, Martin-Subero JI, Siegmund D, Muerkoster S, Pagerols-Raluy L, Siebert R, Wajant H, & Kalthoff H: CD95 and TRAF2 promote invasiveness of pancreatic cancer cells. Faseb J 2005; 19:620-622.

38. Ikebe M, Kitaura Y, Nakamura M, Tanaka H, Yamasaki A, Nagai S, Wada J, Yanai K, Koga K, Sato N, Kubo M, Tanaka M, Onishi H, & Katano M: Lipopolysaccharide (LPS) increases the invasive ability of pancreatic cancer cells through the TLR4/MyD88 signaling pathway. J Surg Oncol 2009; 100:725-731.

39. Lopes RB, Gangeswaran R, McNeish IA, Wang Y, & Lemoine NR: Expression of the IAP protein family is dysregulated in pancreatic cancer cells and is important for resistance to chemotherapy. Int J Cancer 2007;

40. Bashyam MD, Bair R, Kim YH, Wang P, Hernandez-Boussard T, Karikari CA, Tibshirani R, Maitra A, & Pollack JR: Array-based comparative genomic hybridization identifies localized DNA amplifications and homozygous deletions in pancreatic cancer. Neoplasia 2005; 7:556-562.

41. Giagkousiklidis S, Vellanki SH, Debatin KM, & Fulda S: Sensitization of pancreatic carcinoma cells for gamma-irradiation-induced apoptosis by XIAP inhibition. Oncogene 2007; 26:7006-7016.

42. Esposito I, Kleeff J, Abiatari I, Shi X, Giese N, Bergmann F, Roth W, Friess H, & Schirmacher P: Overexpression of cellular inhibitor of apoptosis protein 2 is an early event in the progression of pancreatic cancer. J Clin Pathol 2007; 60:885-895.

43. Li Y, Jian Z, Xia K, Li X, Lv X, Pei H, Chen Z, & Li J: XIAP is related to the chemoresistance and inhibited its expression by RNA interference sensitize pancreatic carcinoma cells to chemotherapeutics. Pancreas 2006; 32:288-296.

44. Shrikhande SV, Kleeff J, Kayed H, Keleg S, Reiser C, Giese T, Buchler MW, Esposito I, & Friess H: Silencing of X-linked inhibitor of apoptosis (XIAP) decreases gemcitabine resistance of pancreatic cancer cells. Anticancer Res 2006; 26:3265-3273.

45. Karikari CA, Roy I, Tryggestad E, Feldmann G, Pinilla C, Welsh K, Reed JC, Armour EP, Wong J, Herman J, Rakheja D, & Maitra A: Targeting the apoptotic machinery in pancreatic cancers using small-molecule antagonists of the X-linked inhibitor of apoptosis protein. Mol Cancer Ther 2007;

46. Vogler M, Walczak H, Stadel D, Haas TL, Genze F, Jovanovic M, Bhanot U, Hasel C, Moller P, Gschwend JE, Simmet T, Debatin KM, & Fulda S: Small molecule XIAP inhibitors enhance TRAIL-induced apoptosis and antitumor activity in preclinical models of pancreatic carcinoma. Cancer Res 2009; 69:2425-2434.

47. Sagol O, Yavuzsen T, Oztop I, Ulukus C, Ylmaz U, Alakavuklar M, Karademir S, Obuz F, Astarcoglu H, & Astarcoglu I: The effect of apoptotic activity, survivin, Ki-67, and P-glycoprotein expression on prognosis in pancreatic carcinoma. Pancreas 2005; 30:343-348.

48. Qiao JG, Zhang YQ, Yin YC, & Tan Z: Expression of Survivin in pancreatic cancer and its correlation to expression of Bcl-2. World J Gastroenterol 2004; 10:2759-2761.

49. Tonini G, Vincenzi B, Santini D, Scarpa S, Vasaturo T, Malacrino C, Coppola R, Magistrelli P, Borzomati D, Baldi A, Antinori A, Caricato M, Nuzzo G, & Picciocchi A: Nuclear and cytoplasmic expression of survivin in 67 surgically resected pancreatic cancer patients. Br J Cancer 2005; 92:2225-2232.

50. Satoh K, Kaneko K, Hirota M, Masamune A, Satoh A, & Shimosegawa T: Expression of survivin is correlated with cancer cell apoptosis and is involved in the development of human pancreatic duct cell tumors. Cancer 2001; 92:271-278.

51. Lee MA, Park GS, Lee HJ, Jung JH, Kang JH, Hong YS, Lee KS, Kim DG, & Kim SN: Survivin expression and its clinical significance in pancreatic cancer. BMC Cancer 2005; 5:127.

52. Tsuji N, Asanuma K, Kobayashi D, Yagihashi A, & Watanabe N: Introduction of a survivin gene-specific small inhibitory RNA inhibits growth of pancreatic cancer cells. Anticancer Res 2005; 25:3967-3972.

53. Mouratidis PX, Colston KW, & Dalgleish AG: Doxycycline induces caspase-dependent apoptosis in human pancreatic cancer cells. Int J Cancer 2007; 120:743-752.

54. Phillips PA, Dudeja V, McCarroll JA, Borja-Cacho D, Dawra RK, Grizzle WE, Vickers SM, & Saluja AK: Triptolide induces pancreatic cancer cell death via inhibition of heat shock protein 70. Cancer Res 2007; 67:9407-9416.

55. Aghdassi A, Phillips P, Dudeja V, Dhaulakhandi D, Sharif R, Dawra R, Lerch MM, & Saluja A: Heat shock protein 70 increases tumorigenicity and inhibits apoptosis in pancreatic adenocarcinoma. Cancer Res 2007; 67:616-625.

56. Dudeja V, Mujumdar N, Phillips P, Chugh R, Borja-Cacho D, Dawra RK, Vickers SM, & Saluja AK: Heat shock protein 70 inhibits apoptosis in cancer cells through simultaneous and independent mechanisms. Gastroenterology 2009; 136:1772-1782.

57. Attri J, Srinivasan R, Majumdar S, Radotra BD, & Wig J: Alterations of tumor suppressor gene p16INK4a in pancreatic ductal carcinoma. BMC Gastroenterol 2005; 5:22.

58. Moore PS, Sipos B, Orlandini S, Sorio C, Real FX, Lemoine NR, Gress T, Bassi C, Kloppel G, Kalthoff H, Ungefroren H, Lohr M, & Scarpa A: Genetic profile of 22 pancreatic carcinoma cell lines. Analysis of K-ras, p53, p16 and DPC4/Smad4. Virchows Arch 2001; 439:798-802.

59. Qanungo S, Basu A, Das M, & Haldar S: 2-Methoxyestradiol induces mitochondria dependent apoptotic signaling in pancreatic cancer cells. Oncogene 2002; 21:4149-4157.

60. Huang S, Okumura K, & Sinicrope FA: BH3 mimetic obatoclax enhances TRAIL-mediated apoptosis in human pancreatic cancer cells. Clin Cancer Res 2009; 15:150-159.

61. Sclabas GM, Fujioka S, Schmidt C, Evans DB, & Chiao PJ: NF-kappaB in pancreatic cancer. Int J Gastrointest Cancer 2003; 33:15-26.

62. Liao Q, Ozawa F, Friess H, Zimmermann A, Takayama S, Reed JC, Kleeff J, & Buchler MW: The anti-apoptotic protein BAG-3 is overexpressed in pancreatic cancer and induced by heat stress in pancreatic cancer cell lines. FEBS Lett 2001; 503:151-157.

63. Miyamoto Y, Hosotani R, Wada M, Lee JU, Koshiba T, Fujimoto K, Tsuji S, Nakajima S, Doi R, Kato M, Shimada Y, & Imamura M: Immunohistochemical analysis of Bcl-2, Bax, Bcl-X, and Mcl-1 expression in pancreatic cancers. Oncology 1999; 56:73-82.

64. Shi X, Liu S, Kleeff J, Friess H, & Buchler MW: Acquired resistance of pancreatic cancer cells towards 5-Fluorouracil and gemcitabine is associated with altered expression of apoptosis-regulating genes. Oncology 2002; 62:354-362.

65. Boucher MJ, Morisset J, Vachon PH, Reed JC, Laine J, & Rivard N: MEK/ERK signaling pathway regulates the expression of Bcl-2, Bcl-X(L), and Mcl-1 and promotes survival of human pancreatic cancer cells. J Cell Biochem 2000; 79:355-369.

66. Guoan X, Hanning W, Kaiyun C, & Hao L: Adenovirus-mediated siRNA targeting Mcl-1 gene increases radiosensitivity of pancreatic carcinoma cells in vitro and in vivo. Surgery 2010; 147:553-561.

67. Wei SH, Dong K, Lin F, Wang X, Li B, Shen JJ, Zhang Q, Wang R, & Zhang HZ: Inducing apoptosis and enhancing chemosensitivity to gemcitabine via RNA interference targeting Mcl-1 gene in pancreatic carcinoma cell. Cancer Chemother Pharmacol 2008; 62:1055-1064.

68. Evans JD, Cornford PA, Dodson A, Greenhalf W, Foster CS, & Neoptolemos JP: Detailed tissue expression of bcl-2, bax, bak and bcl-x in the normal human pancreas and in chronic pancreatitis, ampullary and pancreatic ductal adenocarcinomas. Pancreatology 2001; 1:254-262.

69. Sharma J, Srinivasan R, Majumdar S, Mir S, Radotra BD, & Wig JD: Bcl-XL protein levels determine apoptotic index in pancreatic carcinoma. Pancreas 2005; 30:337-342.

70. Lee JU, Hosotani R, Wada M, Doi R, Kosiba T, Fujimoto K, Miyamoto Y, Tsuji S, Nakajima S, Nishimura Y, & Imamura M: Role of Bcl-2 family proteins (Bax, Bcl-2 and Bcl-X) on cellular susceptibility to radiation in pancreatic cancer cells. Eur J Cancer 1999; 35:1374-1380.

71. Song JJ, An JY, Kwon YT, & Lee YJ: Evidence for two modes of development of acquired tumor necrosis factor-related apoptosis-inducing ligand resistance. Involvement of Bcl-xL. J Biol Chem 2007; 282:319-328.

72. Okamoto K, Ocker M, Neureiter D, Dietze O, Zopf S, Hahn EG, & Herold C: bcl-2-specific siRNAs restore gemcitabine sensitivity in human pancreatic cancer cells. J Cell Mol Med 2007; 11:349-361.

73. Friess H, Lu Z, Graber HU, Zimmermann A, Adler G, Korc M, Schmid RM, & Buchler MW: bax, but not bcl-2, influences the prognosis of human pancreatic cancer. Gut 1998; 43:414-421.

74. Mohammad RM, Wang S, Banerjee S, Wu X, Chen J, & Sarkar FH: Nonpeptidic small-molecule inhibitor of Bcl-2 and Bcl-XL, (-)-Gossypol, enhances biological effect of genistein against BxPC-3 human pancreatic cancer cell line. Pancreas 2005; 31:317-324.

75. Ohno I, Eibl G, Odinokova I, Edderkaoui M, Damoiseaux RD, Yazbec M, Abrol R, Goddard WA, 3rd, Yokosuka O, Pandol SJ, & Gukovskaya AS: Rottlerin stimulates apoptosis in pancreatic cancer cells through interactions with proteins of the Bcl-2 family. Am J Physiol Gastrointest Liver Physiol 2010; 298:G63-73.

76. Fritsche P, Seidler B, Schuler S, Schnieke A, Gottlicher M, Schmid RM, Saur D, & Schneider G: HDAC2 mediates therapeutic resistance of pancreatic cancer cells via the BH3-only protein NOXA. Gut 2009; 58:1399-1409.

77. Abe T, Toyota M, Suzuki H, Murai M, Akino K, Ueno M, Nojima M, Yawata A, Miyakawa H, Suga T, Ito H, Endo T, Tokino T, Hinoda Y, & Imai K: Upregulation of BNIP3 by 5-aza-2'-deoxycytidine sensitizes pancreatic cancer cells to hypoxia-mediated cell death. J Gastroenterol 2005; 40:504-510.

78. Erkan M, Kleeff J, Esposito I, Giese T, Ketterer K, Buchler MW, Giese NA, & Friess H: Loss of BNIP3 expression is a late event in pancreatic cancer contributing to chemoresistance and worsened prognosis. Oncogene 2005; 24:4421-4432.

79. Okami J, Simeone DM, & Logsdon CD: Silencing of the hypoxia-inducible cell death protein BNIP3 in pancreatic cancer. Cancer Res 2004; 64:5338-5346.

80. Akada M, Crnogorac-Jurcevic T, Lattimore S, Mahon P, Lopes R, Sunamura M, Matsuno S, & Lemoine NR: Intrinsic chemoresistance to gemcitabine is associated with decreased expression of BNIP3 in pancreatic cancer. Clin Cancer Res 2005; 11:3094-3101.

81. Mahon PC, Baril P, Bhakta V, Chelala C, Caulee K, Harada T, & Lemoine NR: S100A4 contributes to the suppression of BNIP3 expression, chemoresistance, and inhibition of apoptosis in pancreatic cancer. Cancer Res 2007; 67:6786-6795.

82. Ishida M, Sunamura M, Furukawa T, Akada M, Fujimura H, Shibuya E, Egawa S, Unno M, & Horii A: Elucidation of the relationship of BNIP3 expression to gemcitabine chemosensitivity and prognosis. World J Gastroenterol 2007; 13:4593-4597.

83. Fok JY & Mehta K: Tissue transglutaminase induces the release of apoptosis inducing factor and results in apoptotic death of pancreatic cancer cells. Apoptosis 2007; 12:1455-1463.

84. Liptay S, Weber CK, Ludwig L, Wagner M, Adler G, & Schmid RM: Mitogenic and antiapoptotic role of constitutive NF-kappaB/Rel activity in pancreatic cancer. Int J Cancer 2003; 105:735-746.

85. Wang W, Abbruzzese JL, Evans DB, Larry L, Cleary KR, & Chiao PJ: The nuclear factor-kappa B RelA transcription factor is constitutively activated in human pancreatic adenocarcinoma cells. Clin Cancer Res 1999; 5:119-127.

86. Sclabas GM, Fujioka S, Schmidt C, Fan Z, Evans DB, & Chiao PJ: Restoring apoptosis in pancreatic cancer cells by targeting the nuclear factor-kappaB signaling pathway with the anti-epidermal growth factor antibody IMC-C225. J Gastrointest Surg 2003; 7:37-43; discussion 43.

87. Kallifatidis G, Rausch V, Baumann B, Apel A, Beckermann BM, Groth A, Mattern J, Li Z, Kolb A, Moldenhauer G, Altevogt P, Wirth T, Werner J, Schemmer P, Buchler MW, Salnikov AV, & Herr I: Sulforaphane targets pancreatic tumour-initiating cells by NF-kappaB-induced antiapoptotic signalling. Gut 2009; 58:949-963.

88. Kong R, Sun B, Jiang H, Pan S, Chen H, Wang S, Krissansen GW, & Sun X: Downregulation of nuclear factor-kappaB p65 subunit by small interfering RNA synergizes with gemcitabine to inhibit the growth of pancreatic cancer. Cancer Lett 2010; 291:90-98.

89. Yip-Schneider MT, Lin A, Barnard D, Sweeney CJ, & Marshall MS: Lack of elevated MAP kinase (Erk) activity in pancreatic carcinomas despite oncogenic K-ras expression. Int J Oncol 1999; 15:271-279.

90. Pellegata NS, Sessa F, Renault B, Bonato M, Leone BE, Solcia E, & Ranzani GN: K-ras and p53 gene mutations in pancreatic cancer: ductal and nonductal tumors progress through different genetic lesions. Cancer Res 1994; 54:1556-1560.

91. Weyrer K, Feichtinger H, Haun M, Weiss G, Ofner D, Weger AR, Umlauft F, & Grunewald K: p53, Ki-ras, and DNA ploidy in human pancreatic ductal adenocarcinomas. Lab Invest 1996; 74:279-289.

92. Basu A, Castle VP, Bouziane M, Bhalla K, & Haldar S: Crosstalk between extrinsic and intrinsic cell death pathways in pancreatic cancer: synergistic action of estrogen metabolite and ligands of death receptor family. Cancer Res 2006; 66:4309-4318.

93. Yamamoto S, Tomita Y, Hoshida Y, Morooka T, Nagano H, Dono K, Umeshita K, Sakon M, Ishikawa O, Ohigashi H, Nakamori S, Monden M, & Aozasa K: Prognostic significance of activated Akt expression in pancreatic ductal adenocarcinoma. Clin Cancer Res 2004; 10:2846-2850.

94. Wang YY & Cui QC: [Recent advances in gene change of pancreatic cancer]. Zhongguo Yi Xue Ke Xue Yuan Xue Bao 2004; 26:79-82.

95. Lee CS & Charalambous D: Immunohistochemical localisation of the c-fos oncoprotein in pancreatic cancers. Zentralbl Pathol 1994; 140:271-275.

96. Gysin S, Lee SH, Dean NM, & McMahon M: Pharmacologic inhibition of RAF-->MEK-->ERK signaling elicits pancreatic cancer cell cycle arrest through induced expression of p27Kip1. Cancer Res 2005; 65:4870-4880.

97. Xue A, Xue M, Jackson C, & Smith RC: Suppression of urokinase plasminogen activator receptor inhibits proliferation and migration of pancreatic adenocarcinoma cells via regulation of ERK/p38 signaling. Int J Biochem Cell Biol 2009; 41:1731-1738.

98. Trauzold A, Schmiedel S, Sipos B, Wermann H, Westphal S, Roder C, Klapper W, Arlt A, Lehnert L, Ungefroren H, Johannes FJ, & Kalthoff H: PKCmu prevents CD95-mediated apoptosis and enhances proliferation in pancreatic tumour cells. Oncogene 2003; 22:8939-8947.

99. Farrow B, Thomas RP, Wang XF, & Evers BM: Activation of conventional PKC isoforms increases expression of the pro-apoptotic protein Bad and TRAIL receptors. Int J Gastrointest Cancer 2002; 32:63-72.

100. Nuevemann D, Christgen M, Ungefroren H, & Kalthoff H: Stable expression of temperature-sensitive p53: A suitable model to study wild-type p53 function in pancreatic carcinoma cells. Oncol Rep 2006; 16:575-579.

101. Yu G, Zhu MH, Zhu Z, Ni CR, Zheng JM, & Li FM: Expression of ATM protein and its relationship with p53 in pancreatic carcinoma with tissue array. Pancreas 2004; 28:421-426.
