# Supplementary material for: Examination of Apoptosis Signaling in Pancreatic Cancer by Computational Signal Transduction Analysis
Source: PLoS One. 2010 Aug 19;5(8):e12243. doi: 10.1371/journal.pone.0012243 (PMC2924379; doi:10.1371/journal.pone.0012243)
Supplement: File S2 — Characteristics of our protein interaction prediction (A). Examples of structural models of three possible new interactions in the cell death pathway (more than 30% sequence and interface identity). The structural alignment between template and interacting protein structures is <2 Angstrom. 1 = Arts-Apollon; 2 = p16-ERK; 3 = p16-JNK (B). (2.16 MB PPT) [file pone.0012243.s002.ppt]

## Slide 1
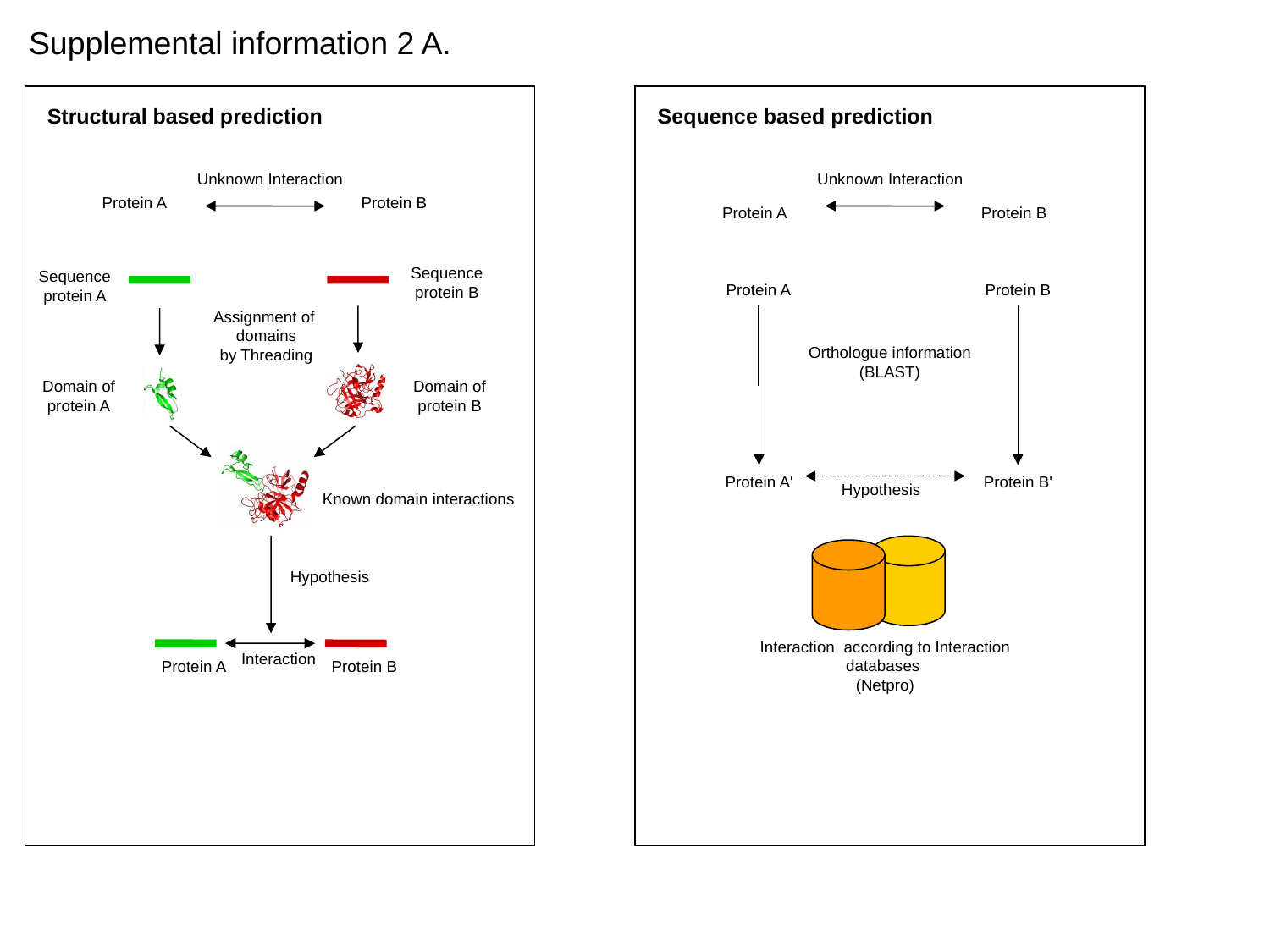

Supplemental information 2 A.
Structural based prediction
Sequence based prediction
Unknown Interaction
Unknown Interaction
Protein A
Protein B
Protein A
Protein B
Sequence protein B
Sequence
protein A
Protein A
Protein B
Assignment of
domains
by Threading
Orthologue information (BLAST)
Domain of
protein A
Domain of
protein B
Protein A'
Protein B'
Hypothesis
Known domain interactions
Hypothesis
Interaction according to Interaction databases
(Netpro)
Protein A
Protein B
Interaction

## Slide 2
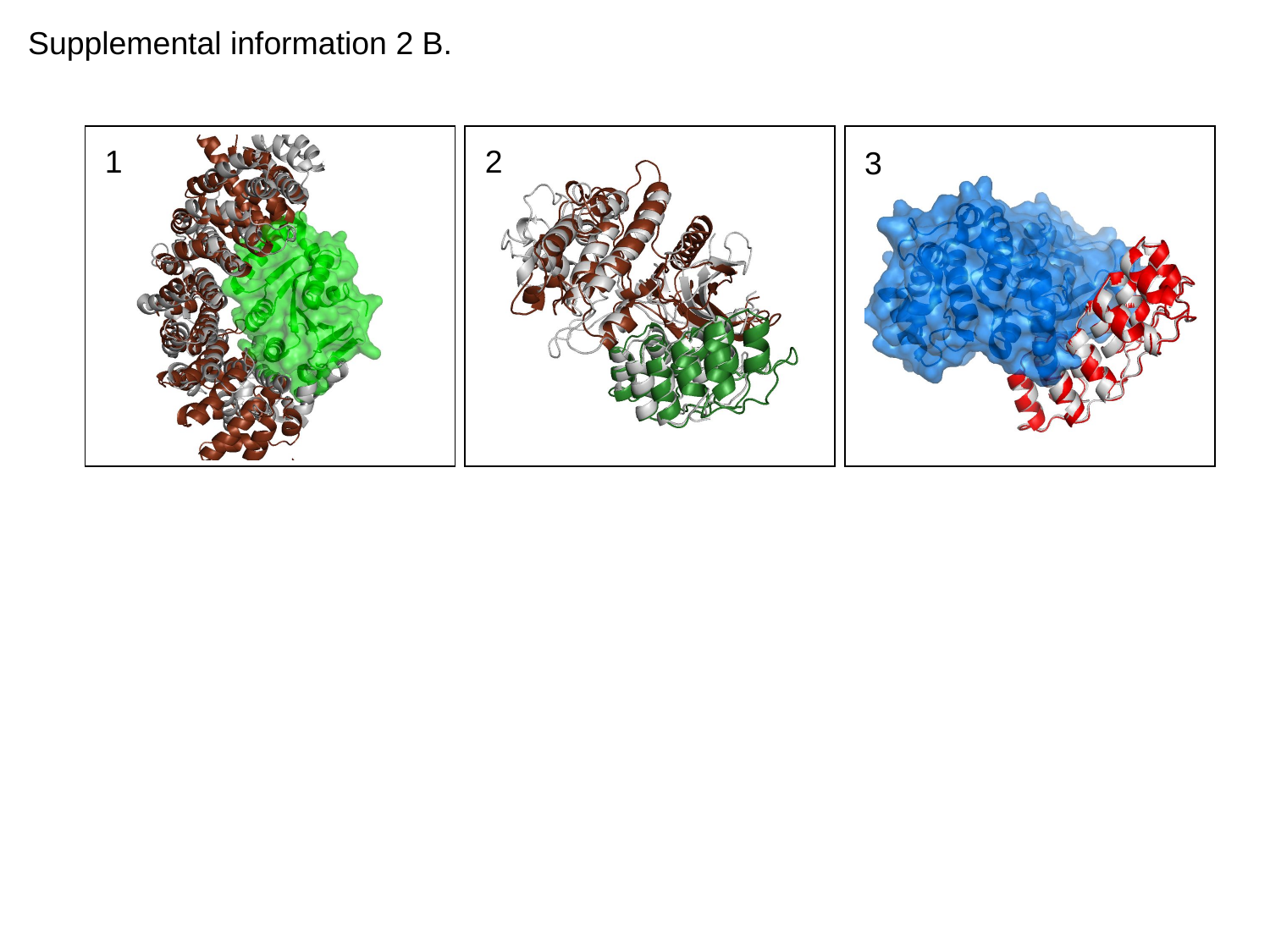

Supplemental information 2 B.
1
2
3
